# Evidence of Lactobacillus strains shared between the female urinary and vaginal microbiota

**DOI:** 10.1099/mgen.0.001267

**Published:** 2024-07-01

**Authors:** Haley Atkins, Baani Sabharwal, Leah Boger, Natalie Stegman, Alexander Kula, Alan J. Wolfe, Swarnali Banerjee, Catherine Putonti

**Affiliations:** 1 Bioinformatics Program, Loyola University Chicago, Chicago, IL, USA; 2 Department of Molecular Environmental Biology, University of California, Berkeley, Berkeley, CA, USA; 3 Data Science Program, Loyola University Chicago, Chicago, IL, USA; 4 Department of Biology, Loyola University Chicago, Chicago, IL, USA; 5 Department of Microbiology and Immunology, Loyola University Chicago, Maywood, IL, USA; 6 Department of Mathematics and Statistics, Loyola University Chicago, Chicago, IL, USA

**Keywords:** connected microbiota, *Lactobacillus*, urogenital, urinary microbiome, vaginal microbiome

## Abstract

*Lactobacillus* species are common inhabitants of the ‘healthy’ female urinary and vaginal communities, often associated with a lack of symptoms in both anatomical sites. Given identification by prior studies of similar bacterial species in both communities, it has been hypothesized that the two microbiotas are in fact connected. Here, we carried out whole-genome sequencing of 49 *Lactobacillus* strains, including 16 paired urogenital samples from the same participant. These strains represent five different *Lactobacillus* species: *L. crispatus*, * L. gasseri*, *L. iners*, *L. jensenii*, and *L. paragasseri*. Average nucleotide identity (ANI), alignment, single-nucleotide polymorphism (SNP), and CRISPR comparisons between strains from the same participant were performed. We conducted simulations of genome assemblies and ANI comparisons and present a statistical method to distinguish between unrelated, related, and identical strains. We found that 50 % of the paired samples have identical strains, evidence that the urinary and vaginal communities are connected. Additionally, we found evidence of strains sharing a common ancestor. These results establish that microbial sharing between the urinary tract and vagina is not limited to uropathogens. Knowledge that these two anatomical sites can share lactobacilli in females can inform future clinical approaches.

**Impact Statement**

Many of the bacteria found within the female urinary tract also inhabit the vagina. As such, prior researchers have hypothesized that these two anatomical sites share a microbiota. Here, we have sequenced isolates of *Lactobacillus* species collected from urine samples and vaginal swabs from the same individual. A statistical method presented here enables us to distinguish between unrelated, related, and identical strains. We found identical strains in the urinary and vaginal samples of 8 of the 16 collections, thus providing evidence that these 2 communities are connected. This connection has direct clinical implications, as many urinary and vaginal infections are associated with decreased abundances of lactobacilli. Therapies aimed at increasing *Lactobacillus* in both anatomical sites may expedite return to a ‘healthy’ microbiota.

## Data Summary

Raw reads and genome assemblies were deposited in the NCBI’s SRA and Assembly databases, respectively. SRA accession numbers are SRR26772071–SRR26772118. Genome assembly accession numbers are JAWWWC000000000–JAWWXW000000000. All code generated as part of this work is publicly available at https://github.com/putonti/LactoCompare.

## Introduction

The female urinary tract and vagina contain many of the same *Lactobacillus* species, including *L. crispatus*, *L. iners*, *L. gasseri*, and *L. jensenii* [1,2]. Communities dominated by lactobacilli are frequently associated with ‘health’ or lack of symptoms [3,4]. *Lactobacillus* strains isolated from female urogenital samples have routinely exhibited antimicrobial properties [5,11], suggesting that they play a role in inhibiting or killing pathogens in the urinary tract and/or vagina. Studies of the healthy (asymptomatic) bladder microbiome have shown that it is frequently predominated by lactobacilli [2]. Likewise, the healthy vaginal community typically consists of >70 % lactobacilli [12], and reduced *Lactobacillus* in the vaginal community often coincides with bacterial vaginosis (BV) [13,15]. While *L. crispatus* has been repeatedly associated with both urinary and vaginal health, *L. iners* is often abundant in females with BV [16,17]. *L. iners*, *L. gasseri*, and *L. jensenii* have frequently been observed within the urinary microbiome of females with incontinence [2,3, 18]. Nevertheless, *L. gasseri* and *L. jensenii* have been found to be effective in inhibiting the growth of several urogenital pathogens [11,19].

Studies where both the urinary tract and vagina were sampled from the same female participant have identified similar genera [20,21] and similar strains [21,22]. This supports the current hypothesis that the urinary and vaginal microbiota are connected. Clinical evidence is consistent with this hypothesis as well. The vagina is thought to play a role in the development of urinary tract infections (UTIs) originating from intestinal microbes [22,23]. Furthermore, studies have identified an association between UTI development and a lack of lactobacilli in the vagina [5,24], and BV is associated with a higher risk of UTI development [25,27]. Treatments aimed at restoring *Lactobacillus* dominance in the vaginal microbiota also have observed a reduction of the incidence of recurrent UTI symptoms [28,29] and an increase in the abundance of *Lactobacillus* species in the urinary tract [30,31].

Building off these previous studies, we explored strains of the same *Lactobacillus* species found in the urinary tract and vagina of the same individual to see if the strains were genotypically similar or the same. In doing so, we can specifically test the hypothesis that the two anatomical sites have a connected microbiota. These strains were isolated as part of a prior study [30] in which samples were collected from postmenopausal females with overactive bladder (OAB) symptoms undergoing oestrogen treatment. The samples were collected prior to treatment and 12 weeks later. We identified participants from this study in which the same species was isolated from both a urine sample (either a voided or catheterized sample) and a vaginal swab sample and sequenced these isolates. Here, we present the genome sequencing and comparative analysis of these genomes representing five *Lactobacillus* species: *L. crispatus*, * L. gasseri*, *L. iners*, *L. jensenii*, and *L. paragasseri*. A statistical method presented here enables us to distinguish between unrelated, related, and identical strains using average nucleotide identity (ANI), all of which were observed in our sample set.

## Methods

### Isolation of strains

**Fig. 1.**
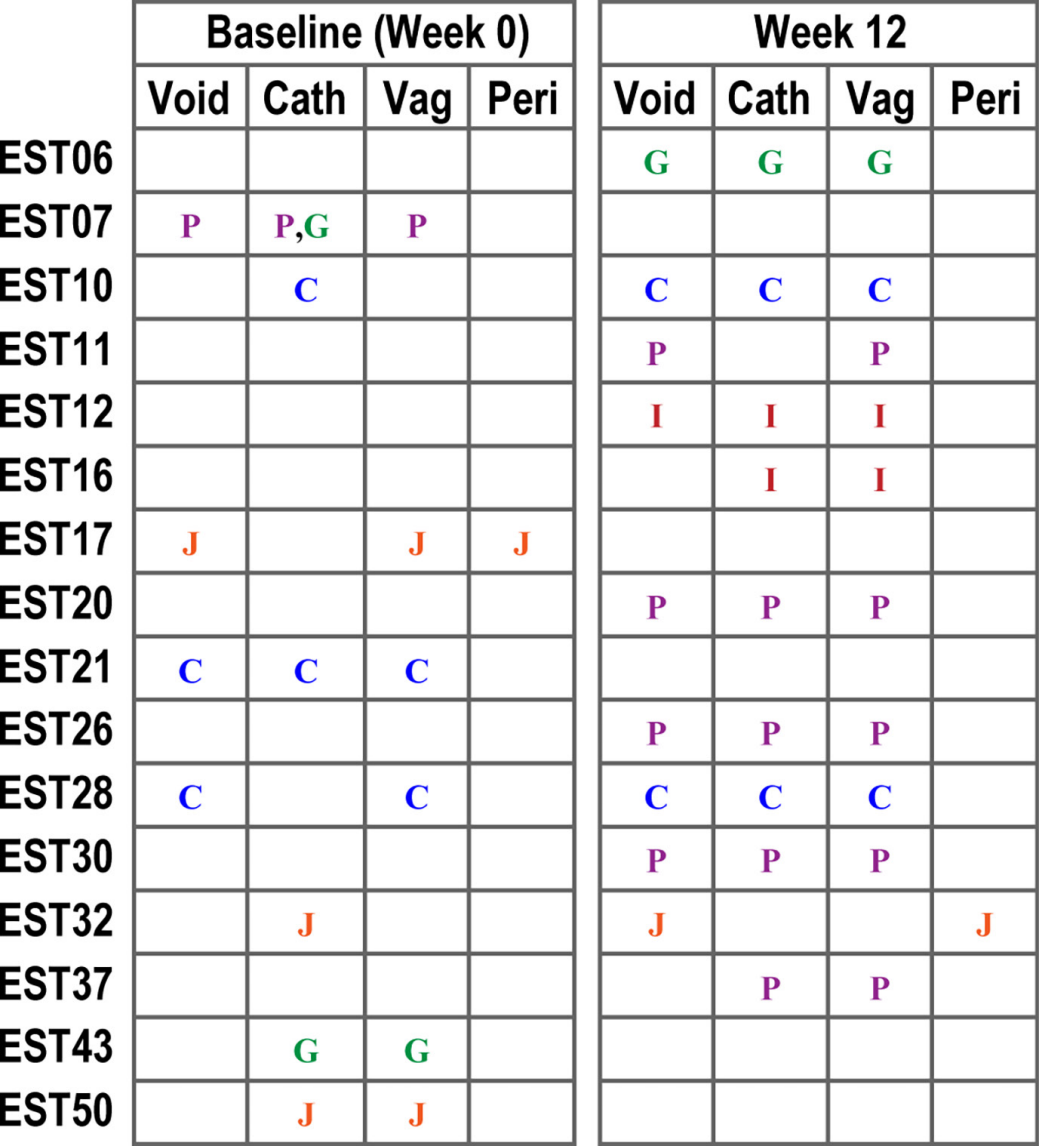
Schematic describing which genomes were generated for the samples collected by participant (EST##), time of collection (baseline or week 12), and sampled site (voided urine, catheterized urine, vaginal swab, perineal swab). C*, L. crispatus*; G, *L. gasseri*; I, *L. iners*; J, *L. jensenii*; *P, L. paragasseri*.

Strains were isolated as part of prior IRB-approved studies (Loyola University Chicago, IRB #207 152 and #207 777). Described in detail in Thomas-White *et al*. [30], samples were collected from females seeking OAB treatment. Briefly, voided and catheterized urine specimens were collected, and strains were isolated by the expanded quantitative urine culture (EQUC) protocol [32]. Vaginal and perineal swabs were collected using a BD ESwab collection kit (catalogue no. 220245). Each swab was swirled in the 1 ml of bacterial preservative (liquid Amies) and an aliquot was provided for EQUC processing. Isolates were identified via MALDI-TOF, as previously described [33], and stored at −80 °C. Fig. 1 visualizes which species were isolated from each participant and sample type.

### DNA extraction and sequencing

Each strain was streaked on a Columbia nalidixic acid (CNA) agar plate and incubated for 24 h at 35 °C with 5 % CO_2_. Then, a single colony was selected and incubated in deMan–Rogosa–Sharpe (MRS) broth supplement with 1 ml l^−1^ Tween 80 (Sigma-Aldrich) and incubated as described above. DNA was extracted from the liquid culture using the Qiagen DNeasy Blood and Tissue Microbial kit following the manufacturer’s protocol for Gram-positive bacteria. DNA concentrations were quantified using a Qubit fluorometer. DNA was sent to SeqCoast Genomics, LLC (Portsmouth, NH USA) for library preparation and sequencing. Samples were prepared for whole-genome sequencing using an Illumina DNA Prep tagmentation kit and unique dual indexes. Sequencing was performed on the Illumina NextSeq2000 platform (San Diego, CA, USA) using a 300-cycle flow cell kit, producing 2×150 bp paired reads. One–two per cent PhiX control was spiked into the run to support optimal base calling. Read demultiplexing, read trimming, and run analytics were performed using DRAGEN v3.10.12 (Illumina). Each sample was sequenced to produce a minimum of 200 Mbp (100× coverage); as Table S1 (available in the online version of this article) shows, this was exceeded for all but one strain (which had a coverage of 94.779×).

### Genome assembly and annotation

The raw reads were trimmed using BBDuk (part of the BBMap suite, v39.01; sourceforge.net/projects/bbmap/) with the following parameters: qtrim=rl, ftl=15, ftr=135, maq=20, maxns=0, and trimq=20. The genomes were assembled via SPades v3.15.4 using the --only-assembler option [34]. Contigs <1000 bp were trimmed from the assembly via a Python script. Final genome assembly quality was verified using CheckM v1.2.2 [35]. We set the threshold for quality assemblies as completeness >95 % and contamination <5 %. One genome, *L. crispatus* UMB4277, included significant contamination (47.2 %) due to the presence of a *Staphylococcus*. Using the web-based tool BV-BRC [36], the raw reads were binned using the metagenomic binning service, identifying the *L. crispatus* genome, which was used for analyses here. CheckM completeness and contamination values were computed by BV-BRC for the *L. crispatus* UMB4277 (EST21 baseline voided urine) genome sequence assembled here as 99.8 and 0.5 %, respectively. Deposited genomes were annotated by the NCBI Prokaryotic Genome Annotation Pipeline (PGAP) [37]. (*L. crispatus* UMB4277 was annotated with PGAP v6.7; all other genomes were annotated with v6.6.)

### Comparative genomics

Genome assemblies were compared via average nucleotide identity (ANI) using the tool FastANI v1.32 [38] with default parameters (k=16). Next, genome assemblies were aligned using the progressiveMauve algorithm through the Mauve plug-in [39] in Geneious Prime (v2023.1.2; Dotmatics, Auckland, NZ), and the assemblies were exported to file. The number of single-nucleotide polymorphisms (SNPs) was calculated from the assemblies via a Python script available at the project repository (https://github.com/putonti/LactoCompare).

Pangenomes were developed for each species using anvi’o v7.1 [40]. Databases for each species were generated using the *anvi-gen-contigs-database* command and annotated using the hmms and cogs databases with the *anvi-run-hmms* and *anvi-run-ncbi-cogs* commands. Pangenome analysis was performed using the *anvi-pan-genome* command with the Markov cluster (MCL) inflation parameter set to 8, specifying the use of NCBI BLAST for calculating amino acid similarity among genomes. For each species, the amino acid sequences of the single-copy-number core genome were retrieved using the *anvi-get-sequences-for-gene-clusters* command. The max-num-genes-from-each-genome parameter was set to 1, and the min-num-genomes-gene-cluster-parameter was equal to the number of genomes for the given species. The genomes were then imported into Geneious Prime and aligned with the MAFFT v7.490 plug-in [41]. The phylogenetic trees were derived using the Geneious Tree Builder and visualized using iTOL v6.81 [42].

Genome assemblies were examined for CRISPR arrays using the webtool CRISPRCasFinder with default parameters [43]. For each query, spacer sequences were retrieved and formatted. Spacer sequences were compared between genome assemblies using a Python script available at the project repository. Because these are genome assemblies (i.e. represented via multiple contigs), some spacer arrays were split across contigs and/or identified on different strands between assemblies. Our Python script considered both strands and spacer order split across contigs.

### Statistical analysis

To simulate genome assemblies from the same strain, the fastq files were randomly subsampled using seqtk (https://github.com/lh3/seqtk) and its *sample* command. We randomly subsampled 150 000 read pairs from each R1 and R2 fastq file 50 times. The seqtk *sample* command was seeded using 50 unique seed values generated via the Python random library. Thus, 50 pairs of subsampled reads were produced for each strain. Next, each subsampled fastq pair was trimmed and assembled, as described previously. ANI calculations were then performed for the 25 pairs of simulated genome assemblies. To avoid biasing our simulations, each simulated assembly was only compared to one other simulated assembly.

For each species of *Lactobacillus,* ANI values were pooled together and bootstrapped with replacement 10 000 times. Analysis of variance (ANOVA) was performed to test for strain level variation. We used kernel density estimation to construct an empirical distribution of ANI values for each species. The pairwise ANI values of genome assemblies (Table S1) were projected onto the empirical distribution of ANI values for that species. The null hypothesis tested is that the strains are statistically the same. This was tested against the one-sided alternative that the observed ANI value is an outlier on the left tail of this distribution, i.e. the ANI value corresponds to two dissimilar strains. To test this hypothesis, an empirical *P* value was calculated by estimating the empirical probability using the *pemp* function (EnvStats) in R.

All code for subsampling, written in Python, and statistical analysis, written in R, is available at the project repository.

## Results

Of the 62 participants from the Thomas-White *et al*. study [30], we identified participants for which the same *Lactobacillus* species was isolated from both a urine sample and vaginal swab. In total, 16 participants were selected as the same species had been isolated from more than 1 sampled site. Except for one participant, EST32 12 week, these included isolates from urine and vaginal swab samples. The Thomas-White study included sample collection at ‘baseline’ (week 0) and 12 weeks after enrolment. For some participants, the same species was isolated during the week 0 collection, for others the week 12 collection (Fig. 1). We were able to generate *Lactobacillus* genome sequences for 49 isolates (Tables 1 and S1). Paired (urine+another urogenital site) assemblies were produced for 17 of these unique collections. Our genomes (Tables 1 and S1) include three unpaired samples [*L. crispatus* EST10 baseline (UMB2389), *L. gasseri* EST10 baseline (UMB2385), and *L. jensenii* EST32 baseline (UMB5669)], as we were unable to isolate *Lactobacillus* from the freezer stock. Furthermore, we were unable to isolate the *L. jensenii* from the freezer stock of the vaginal swab from participant EST32’s 12 week collection; thus, this collection only contains a strain collected from a perineal swab (UMB6506) and voided urine (UMB6491). These five genomes, however, were kept for downstream analyses, as they represent genetic diversity within these niches.

**Table 1.** Number of strains per species from each sample type collected

	No. of strains	Urine		Perineal	Vaginal
		Void	Catheter	Swab	Swab
***L. crispatus***	**12**	4	4	0	4
***L. gasseri***	**6**	1	3	0	2
***L. iners***	**5**	1	2	0	2
***L. jensenii***	**8**	2	2	2	2
***L. paragasseri***	**18**	5	7	0	6

### Distinct strain identification

**Fig. 2.**
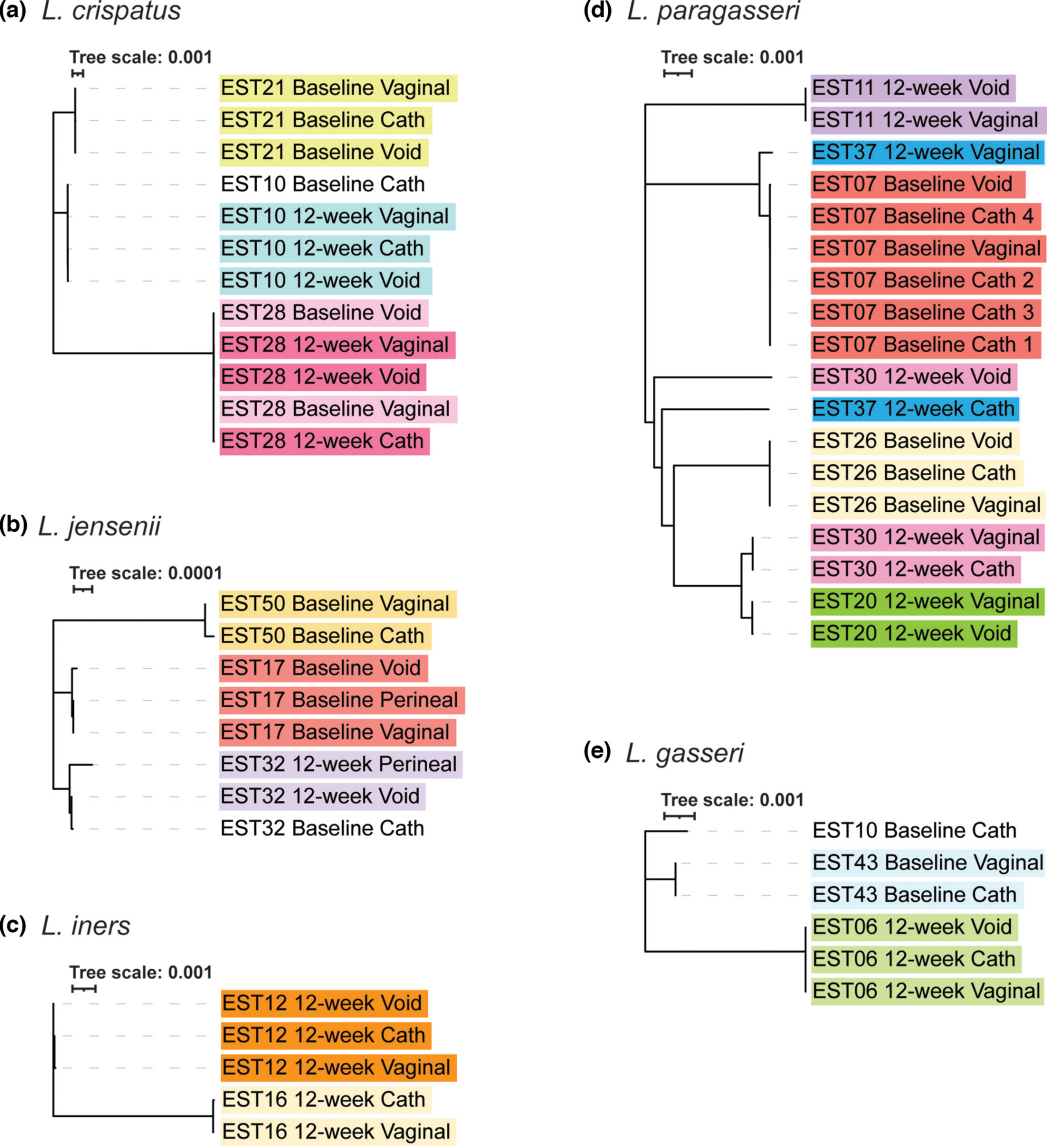
Phylogenomic trees of the single-copy-number core genomes for the five species (**a–e**). For each tree, strains isolated from the same sample (same participant and same time point) are highlighted in the same colour; strains that do not have another strain isolated from this same sample are not highlighted. Strain names are listed as follows: the participant ID (e.g. EST21), the study collection time point (either ‘baseline’ or ‘12 week’), and the sample type [‘vaginal’ (vaginal swab), ‘cath’ (catheterized urine), ‘perineal’ (perineal swab), or ‘void’ (voided urine)].

The single-copy-number core genome for each of the five species was computed and phylogenetic trees were derived to visualize the sequence similarity between strains of the same species that were isolated from the same participant and between participants. Fig. 2 includes the five trees for each of the species represented here. In each tree, strains isolated from the same participant are displayed in the same colour. As these trees show, nearly all strains isolated from the same participant’s collection (same colour) are more similar to each other than they are to strains collected from other participants. There are two exceptions, both among the *L. paragasseri* strains (Fig. 2d). The strains from EST37 12 week are found in two different clades of the tree (Fig. 2d, blue). Similarly, the strain from the EST30 12 week voided urine sample is distinct from those collected from the vaginal swab and catheterized urine sample (Fig. 2d, pink).

To further examine these genomes, we computed pairwise average nucleotide identity (ANI) between genomes from the same collection. Most average ANI values exceeded 99.97 % (Table S2), well above the 95 % used for delineating species [38] and often above the thresholds previously suggested for deciding that two strains are identical (99.97 % or above [44]). Exceptions to this observation are *L. paragasseri* EST37 12 week (Fig. 2d, blue; 98.1906 %) and *L. paragasseri* EST30 12 week (Fig. 2d, pink; 98.9643 %). To complement our ANI analysis, we next conducted whole-genome multiple sequence alignments. Average pairwise genome sequence identity exceeded 96.3 % for all collections examined (Table S2). The greatest number of SNPs between these alignments was determined for the *L. paragasseri* EST30 12 week (1.9883 %), *L. paragasseri* EST37 12 week (2.1583 %), and *L. crispatus* EST28 12 week (2.6277 %) collections (Table S2).

We also identified CRISPR arrays within the genome assemblies, as these arrays provide a history of past phage infections [45]. We assume that identical strains and closely related strains will have the same CRISPR arrays, while unrelated or more distantly related strains may have different CRISPR arrays. All but 11 of the genomes examined contain CRISPR arrays (File S1); the *L. gasseri* genomes in this collection did not contain a CRISPR–Cas system. For 10 of the samples, the same CRISPR array was found in all genomes from the sample. The strains of four samples, *L. jensenii* EST17 baseline, *L. jensenii* EST32 12 week, *L. paragasseri* EST37 12 week, and *L. paragasseri* EST30 12 week, had different CRISPR arrays. Table S3 summarizes the CRISPR analysis, as well as the differences in CRISPR arrays identified in these four samples.

### Distinguishing between identical and similar strains

As the comparative genomic analyses presented thus far illustrate, it is far easier to identify different strains than it is to distinguish between similar and identical strains. In an effort to make such a distinction, we first performed simulations, producing genome assemblies from subsampled reads from the raw fastq files of our sequencing. As detailed in the Methods, we used these simulations to create a distribution of ANI values that could be the result of comparing the same strain. Each species was considered independently, as we were initially uncertain if this distribution of ANI values would vary between species. ANOVA showed significant (*P*<0.05) strain level variation for the same species, thereby signifying that genetic diversity is represented within the distribution.

**Fig. 3.**
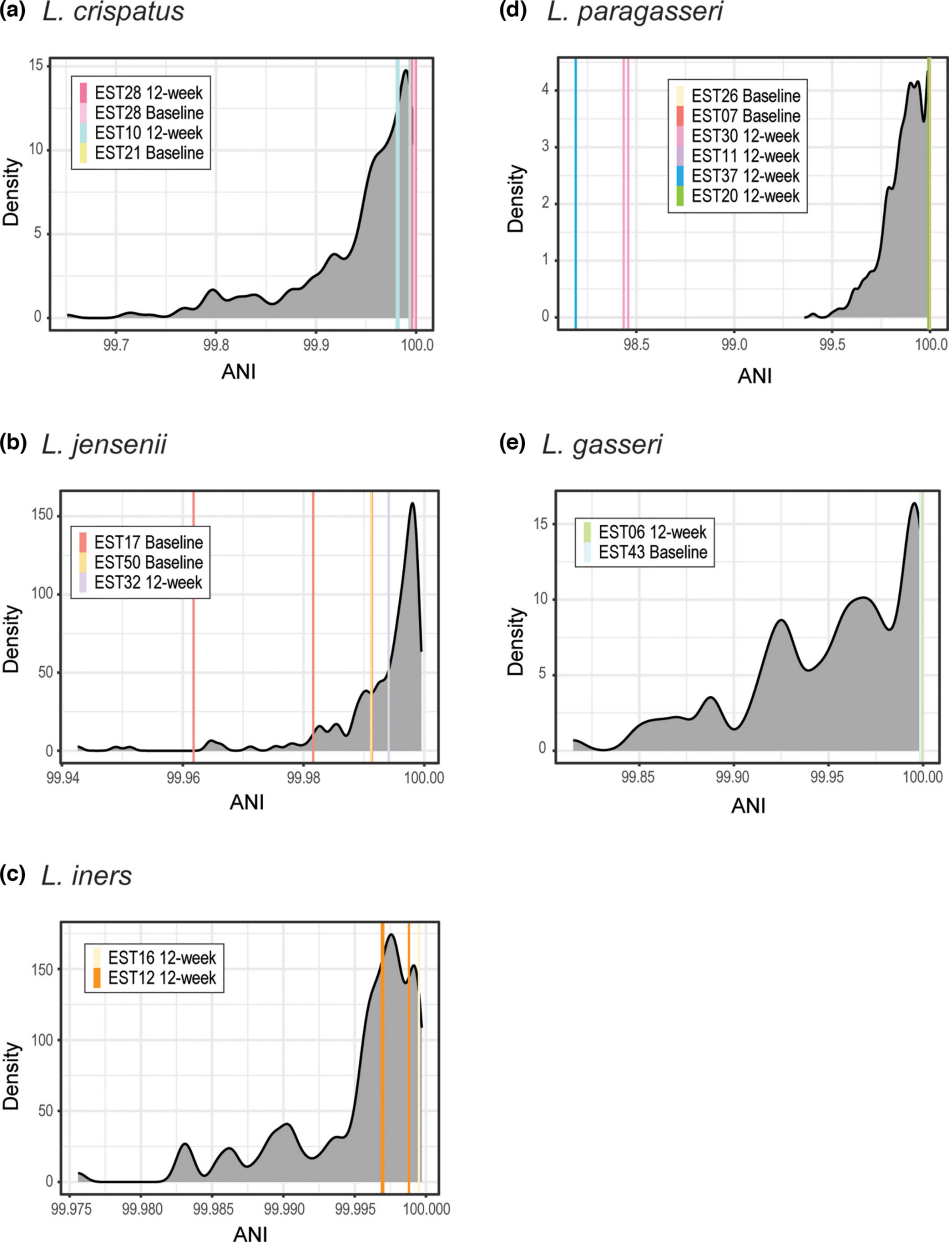
Pairwise ANI values for genomes collected for the same participant and collection time mapped against the distribution of ANI values from pairwise ANI calculations of simulated genomes (shown in grey). Each species is shown independently (**a–e**). Genomes from the same participant/time are the same colour, corresponding with their colour in Fig. 2. The *x*-axis is dependent upon the distribution and ANI values from pairwise genome comparisons.

Using the distributions created from the simulated genome assemblies, we compared all pairwise ANI calculations between the final genome assemblies (assembled from the full fastq data) used in our prior analyses. Fig. 3 shows the distribution of ANI values from our simulations and the pairwise ANI values for each sample’s genome. While most samples have pairwise ANI values within the distribution, strains from the *L. paragasseri* EST37 12 week and *L. paragasseri* EST30 12 week samples do not (Fig. 3d, pink and blue lines), confirming our prior observations (Fig. 2), and suggesting that these are different strains in the different anatomical sites.

Using these distributions, we next computed the empirical *P* values to quantify the confidence in claims that two strains are identical (our null hypothesis). The empirical *P* values among strains from the same sample, as well as empirical *P* values for strains from different samples, are listed in Table S4. Comparisons of genomes between participants are not statistically significant, with empirical *P* values <0.05. Examination of the empirical *P* values from pairwise genome comparisons from the same sample revealed a range of values (Table 2). With empirical *P* values of 1, we can confidently state that the two *L. gasseri* samples are in fact identical. Similarly, the *L. crispatus* strains from the voided urine sample and the vaginal swab sample collected from participant EST28 at 12 week are identical (empirical *P* value=1). Taking a conservative approach in drawing this line between identical and related, we chose the threshold of empirical *P* values ≥0.95 identifying five additional samples containing identical strains: *L. crispatus* EST28 baseline (voided urine and vaginal swab), *L. iners* EST16 12 week (catheterized urine and vaginal swab), *L. paragasseri* EST26 baseline (voided urine and catheterized urine), *L. paragasseri* EST07 baseline (voided urine, catheterized urine, and vaginal swab), and *L. paragasseri* EST30 12 week (catheterized urine and vaginal swab). Thus, in total, eight of the samples contain instances of identical strains – seven of which include identical strains isolated from both the urinary and vaginal microbiota.

**Table 2.** Range of empirical *P* values calculated for ANI values of genomes from the same sample

Species	Participant ID	Study collection time point	No. of genomes analysed	Empirical *P* value ranges
*L. crispatus*	EST10	12 week	3	0.6847–0.8990
*L. crispatus*	EST21	Baseline	2	0.9025
*L. crispatus*	EST28	12 week	3	0.9121–1.0
*L. crispatus*	EST28	Baseline	2	0.9852
*L. gasseri*	EST06	12 week	3	1.0
*L. gasseri*	EST43	Baseline	2	1.0
*L. iners*	EST12	12 week	3	0.5302–0.8097
*L. iners*	EST16	12 week	2	0.9759
*L. jensenii*	EST17	Baseline	3	0.01872–0.2564
*L. jensenii*	EST32	12 week	2*	0.365
*L. jensenii*	EST50	Baseline	2	0.2549
*L. paragasseri*	EST07	Baseline	6	0.9405–0.9892
*L. paragasseri*	EST11	12 week	2	0.9414
*L. paragasseri*	EST20	12 week	2	0.88176
*L. paragasseri*	EST26	Baseline	3	0.91198–0.9629
*L. paragasseri*	EST30	12 week	3	0.0020–0.9992
*L. paragasseri*	EST37	12 week	2	0.002

*Contains strains from voided urine and a perineal swab; vaginal swab not included.

**Fig. 4.**
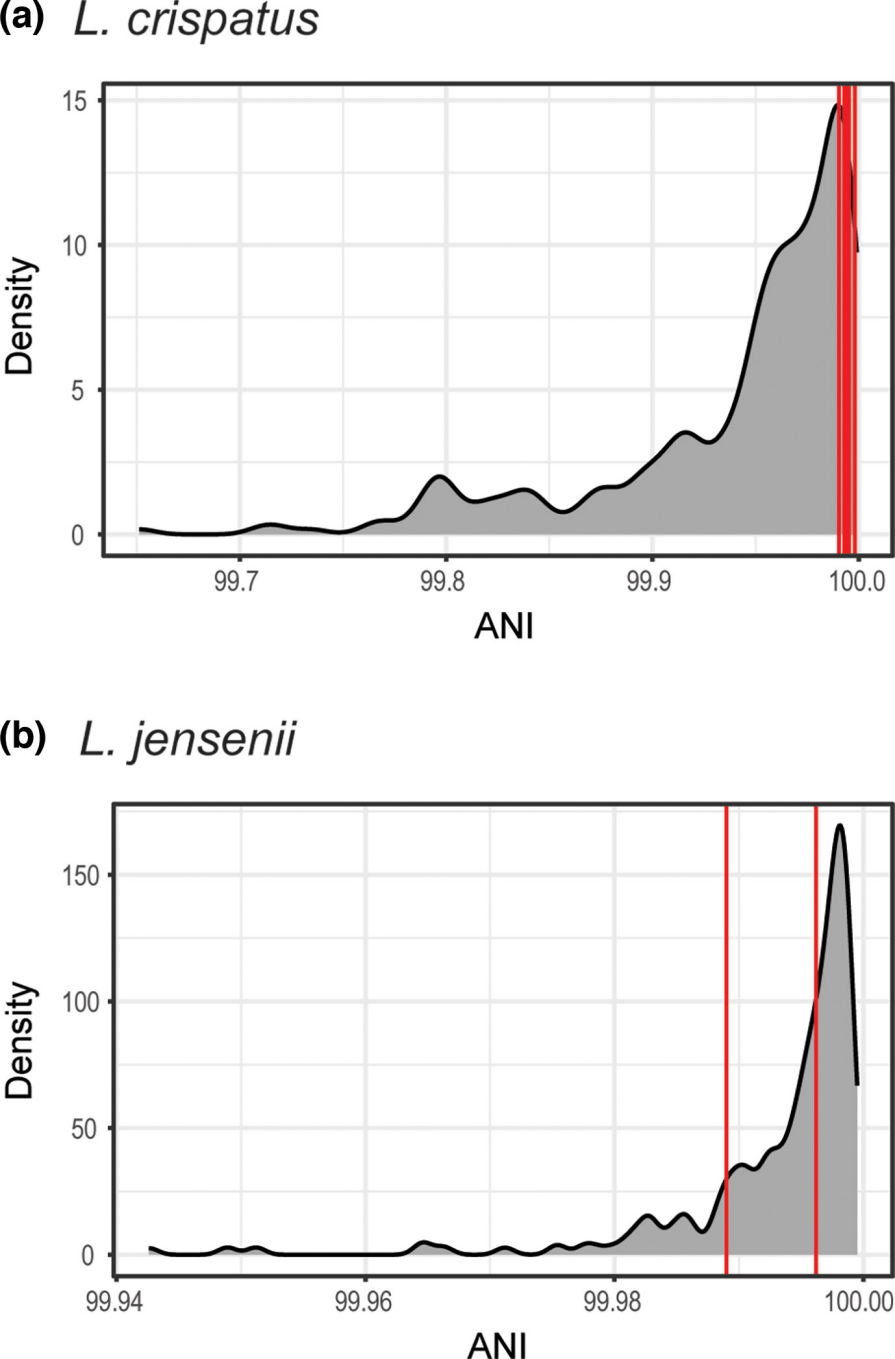
Pairwise ANI values for genomes collected for the same participant at different collection times (in red) mapped against the distribution of ANI values from pairwise ANI calculations of simulated genomes (shown in grey). (**a**) *L. crispatus* comparisons of genomes from EST28 baseline void and vagina and the genomes from EST28 12 week vagina, void, and cath. (**b**) *L. jensenii* comparisons of the genome from EST32 baseline cath and the genomes from EST32 12 week perineal and void. The *x*-axis is dependent upon the distribution and ANI values from pairwise genome comparisons. ANI values can be found in Table S4.

For two of the participants, we had strains from both the baseline time point and the 12 week time point: *L. crispatus* EST28 (baseline, *n*=2; 12 week, *n*=3) and *L. jensenii* EST32 (baseline, *n*=1; 12 week, *n*=2). The ANI values of genomes from different collection time points were superimposed on the distribution of ANI values from our simulations (Fig. 4). Based upon these comparisons, we believe that the *L. crispatus* baseline vaginal strain is related to and likely the ancestor of all three strains from the collection at 12 weeks (empirical *P*>0.9273) (Fig. 4a and Table S4). In contrast, the *L. jensenii* strains from the perineal swab and voided urine sample of participant EST32 at 12 weeks are likely not related to the strain collected from catheterized urine at baseline. EST32 baseline cath vs EST32 12 week perineal has an empirical *P*=0.1743 and EST32 baseline cath vs EST32 12 week void has an empirical *P*=0.5238 (Fig. 4b and Table S4).

## Discussion

While clinical evidence suggests that the urinary and vaginal microbiota can affect each other, here we provide the first evidence that identical strains can be found in both anatomical sites. These results validate the prior hypothesis that the two microbiotas are connected [21]. Although these *Lactobacillus* species are non-motile [46], they may be able to move between these two sites via ‘hitchhiking’ on motile members of these communities [47]. If exchange between the urinary tract and vagina is common, then infections in one must take the other into consideration.

While distinctly different strains are easy to identify by a variety of methods, including ANI, whole-genome alignment, and SNP analysis, distinguishing between strains that share a common ancestor and those that are identical is challenging. If the genome sequences share 100 % nucleotide identity, such a call can easily be made. While lactobacilli reproduce asexually, small variations can arise in their nucleotide sequences [48]. Although prior investigation of *Lacticaseibacillus casei* found the rate of *de novo* mutations to be both low and stable [49], experimental work is needed to ascertain if this is true for species of *Lactobacillus*. Furthermore, nominal nucleotide variations are likely to arise from sequencing and/or assembling reads. Here, we consider such instances in which nominal genome differences are observed as members of the same strain.

Determining a means to distinguish between related and identical strains has yet to be uniformly agreed upon. Single-nucleotide variants (SNVs) between marker genes have frequently been used to distinguish between identical strains and different strains. StrainPhlAn characterizes two genomes as representing different strains if >0.1 % of nucleotides in species-specific marker sequences are polymorphic [50]. However, as an example, in a genomic epidemiologic study of *Klebsiella pneumoniae*, SNV thresholds were misleading, with significant false-positive and false-negative transmission inferences [51]. Alternatively, an ANI threshold (or a variation on the standard ANI measure) is often used to make such a distinction, although a range of values have been used [44,52, 53]. The ANI threshold to distinguish between strains, however, has yet to be determined. A study published earlier this year posed an ANI threshold of >99.99 % based upon their analysis of sequencing *Salinibacter ruber* from two solar saltern sites and their knowledge of the diversity expected [54]. The statistical method used here enables us to quantify our confidence in claims that strains are unrelated, related, or identical based on ANI.

As a proof-of-concept for this approach, we built the species distributions using only the sequence read data generated as part of this study rather than retrieving all publicly available data and performing quality control on that data. Because our ANOVA is statistically significant, we are confident that the distribution is robust, with sufficient genetic diversity represented for each species of interest. In addition to the three collections with pairwise urinary and vaginal strains producing an empirical *P* value of 1 (Table 2), we identified an additional five urine–vaginal collections that are identical. While here we used the empirical *P* value of ≥0.95 to make this determination, further investigation is needed to determine a threshold. Nevertheless, when looking at the pairwise ANI values, it is on par with the most conservative ANI thresholds proposed [44,53], and it is supported by our CRISPR analyses (Table S3). However, there are pairwise comparisons with an empirical *P*-value below this threshold that are still within the distribution of ANI values from our simulations (Fig. 3). This observation highlights the challenge in distinguishing between related and identical strains. Thus, we conclude that 8 of the 16 collections that include strains from both the urinary tract and vagina contain identical strains, evidence that the urinary and vaginal communities are connected.

It should be noted that not all strains from these eight collections were identical, highlighting instances of multiple strains colonizing the urinary tract and/or vagina. There are two strains of *L. paragasseri* from participant EST30’s 12 week collection in the urinary tract, one shared with the vaginal community and the other unique to the voided sample (empirical *P* value=0.002; Table S4). Prior studies have shown that multiple strains of the same species can persist in the vagina [53,55, 56] and urinary tract [57,59]. For all of the samples, with the exception of *L. paragasseri* EST07 baseline catheterized urine samples, only a single colony was selected for the species from the sample and subsequently sequenced. Therefore, we cannot conclusively state that the same or closely related strains did not inhabit these niches, only that they were not selected during processing.

While the urinary–vaginal pairs of three collections are significantly different (empirical *P* value <0.05), six collections (*L. crispatus* EST10 12 week, *L. crispatus* EST21 baseline, *L. iners* EST12 12 week, *L. jensenii* EST50 baseline, *L. paragasseri* EST11 12 week, and *L. paragasseri* EST20 12 week) have empirical *P* values such that the null hypothesis, that the strains are the same, cannot be rejected. We hypothesize that they are related, sharing a common ancestor, further supported by the fact that the strains have identical CRISPR arrays (Table S3). The common ancestry suggests that one site was the source and has subsequently evolved in the other anatomical site. The prevailing hypothesis for UTI development within females, the fecal–perineal–urethral hypothesis, indicates that the vagina is a reservoir prior to colonizing the urinary tract (see review [24]), implying that the urinary strains from these six collections may have evolved from their vaginal ancestors.

Within the vaginal community, strain evolution over time has been observed [53,60]. To date, temporal studies of the urinary microbiome that capture constituents at the strain level are few and short-term, from weeks [61] to a few months [62]. While we only had two species that were found in the same participant at the baseline and 12 week time points, we did find strong evidence of the strains deriving from the same ancestor – most notably, the *L. crispatus* EST28 baseline vaginal strain and the urinary and vaginal strains isolated at 12 weeks. Therefore, the results suggest that the vagina was the source of the urinary strain. Given that the study from which these samples were collected was testing the use of vaginal oestrogen cream for OAB symptoms [30], this treatment process may have facilitated exchange. Furthermore, comparisons of these two time points also provides evidence of strain persistence in the vaginal microbiota. Previous research has found that strains can persist within the vagina for up to a year [53]. Future long-term studies into strain persistence within the urinary tract are needed. Likewise, additional studies considering asymptomatic (‘healthy’) individuals are needed to further our understanding of the interconnectedness of these two microbiota.

## Conclusions

Individuals harbouring the same strain and related strains in both the urinary tract and vagina provide clear evidence of the connection between these two communities. As many lower urinary tract symptoms, UTIs, and BV are associated with decreased abundances of lactobacilli, strategies aimed at increasing lactobacilli in both sites may expedite a return to a *Lactobacillus*-dominated community and thus resolution of symptoms/infection. Additional studies are needed to ascertain if translocation of other urogenital species is common, including what factors facilitate this exchange.

## supplementary material

10.1099/mgen.0.001267 Uncited Supplementary Material 1.

## Abbreviations

ANI: average nucleotide identity
BV: bacterial vaginosis
CNA: Columbia nalidixic acid
EQUC: expanded quantitative urine culture
MCL: markov cluster
MRS: deMan-Rogosa-Sharpe
OAB: overactive bladder
PGAP: prokaryotic genome annotation pipeline
SNP: single nucleotide polymorphism
UTI: urinary tract infection
