# Supplementary material for: Evidence of Lactobacillus strains shared between the female urinary and vaginal microbiota
Source: Microb Genom. 2024 Jul 1;10(7):001267. doi: 10.1099/mgen.0.001267 (PMC11316553; doi:10.1099/mgen.0.001267)
Supplement: Uncited Supplementary Material 1. [file mgen-10-01267-s001.pdf]

Supplementary Table 1. Metadata about the sequenced isolates.

		Sample Metadata					Genome Assembly Metadata						
**Species**	**Strain**	**Participant ID**	**Study Collection Timepoint**	**Date of Collection**	**Collection Site**	**Sample Type**	**BioSample Accession**	**No. of Contigs**	**N50 (bp)**	**Genome Length (bp)**	**Genome Coverage (x)**	**CheckM Contamination**	**CheckM Completeness**
Lactobacillus crispatus	UMB4313	EST10	12-week	2016-06-22	urinary tract	urine (catheter)	SAMN38148620	195	19276	2176756	157.116	0	99.8
Lactobacillus crispatus	UMB4315	EST10	12-week	2016-06-22	vagina	swab	SAMN38148619	214	19451	2198803	193.064	0	99.5
Lactobacillus crispatus	UMB4340	EST10	12-week	2016-06-22	urinary tract	urine (void)	SAMN38148621	237	18845	2248929	137.717	0	99.8
Lactobacillus crispatus	UMB2389	EST10	Baseline	2016-02-22	urinary tract	urine (catheter)	SAMN38148657	197	19276	2176420	210.113	0	99.5
Lactobacillus crispatus	UMB4253	EST21	Baseline	2016-06-20	vagina	swab	SAMN38148642	258	17243	2376708	125.337	0.2	99.8
Lactobacillus crispatus	UMB4352	EST21	Baseline	2016-06-20	urinary tract	urine (catheter)	SAMN38148641	275	17159	2361162	223.987	0.5	99.8
Lactobacillus crispatus	UMB4277	EST21	Baseline	2016-06-20	urinary tract	urine (void)	SAMN41062563	240	17212	2322368	103.1	0.5	99.8
Lactobacillus crispatus	UMB6018	EST28	12-week	2016-12-09	urinary tract	urine (catheter)	SAMN38148638	51	125708	2089588	221.949	0.7	100
Lactobacillus crispatus	UMB6021	EST28	12-week	2016-12-09	vagina	swab	SAMN38148637	35	125706	2056972	174.474	0.9	100
Lactobacillus crispatus	UMB6035	EST28	12-week	2016-12-09	urinary tract	urine (void)	SAMN38148636	36	125708	2055669	136.051	0.9	100
Lactobacillus crispatus	UMB5393	EST28	Baseline	2016-09-09	urinary tract	urine (void)	SAMN38148654	37	125708	2053189	226.178	0.7	100
Lactobacillus crispatus	UMB5409	EST28	Baseline	2016-09-09	vagina	swab	SAMN38148653	36	125706	2058977	186.802	0.7	100
Lactobacillus gasseri	UMB3275	EST06	12-week	2016-05-12	urinary tract	urine (catheter)	SAMN38148612	13	357108	1792286	207.264	1.2	100
Lactobacillus gasseri	UMB3277	EST06	12-week	2016-05-12	vagina	swab	SAMN38148611	15	334068	1790024	177.215	1.2	100
Lactobacillus gasseri	UMB3290	EST06	12-week	2016-05-12	urinary tract	urine (void)	SAMN38148610	15	334068	1790191	194.283	1.2	100
Lactobacillus gasseri	UMB2385	EST10	Baseline	2016-02-22	urinary tract	urine (catheter)	SAMN38148656	61	74844	1898261	221.103	0	100
Lactobacillus gasseri	UMB6820	EST43	Baseline	2017-03-09	urinary tract	urine (catheter)	SAMN38148635	75	46851	1945064	185.535	0	99.7
Lactobacillus gasseri	UMB6827	EST43	Baseline	2017-03-09	vagina	swab	SAMN38148634	75	46851	1945742	169.366	0	99.7
Lactobacillus iners	UMB4066	EST12	12-week	2016-06-16	urinary tract	urine (catheter)	SAMN38148645	18	126004	1300306	247.309	1.1	99.5
Lactobacillus iners	UMB4068	EST12	12-week	2016-06-16	vagina	swab	SAMN38148644	17	126047	1301143	293.101	1.1	99.5
Lactobacillus iners	UMB4096	EST12	12-week	2016-06-16	urinary tract	urine (void)	SAMN38148643	20	118363	1303044	218.122	1.2	99.5
Lactobacillus iners	UMB5013	EST16	12-week	2016-08-04	urinary tract	urine (catheter)	SAMN38148640	12	247419	1293834	208.493	0.9	99.6
Lactobacillus iners	UMB5018	EST16	12-week	2016-08-04	vagina	swab	SAMN38148639	16	247161	1329424	260.695	1.1	99.6
Lactobacillus jensenii	UMB3451	EST17	Baseline	2016-05-20	vagina	swab	SAMN38148627	51	67945	1699743	236.816	0	100
Lactobacillus jensenii	UMB3466	EST17	Baseline	2016-05-20	perineal	swab	SAMN38148628	45	87746	1679746	94.779	0	100
Lactobacillus jensenii	UMB3478	EST17	Baseline	2016-05-20	urinary tract	urine (void)	SAMN38148629	50	67945	1666532	331.874	0	100
Lactobacillus jensenii	UMB6491	EST32	12-week	2017-01-26	urinary tract	urine (void)	SAMN38148630	46	109906	1630286	302.995	0	100
Lactobacillus jensenii	UMB6506	EST32	12-week	2017-01-26	perineal	swab	SAMN38148631	41	114249	1629779	345.926	0	100
Lactobacillus jensenii	UMB5669	EST32	Baseline	2016-10-27	urinary tract	urine (catheter)	SAMN38148655	41	109906	1628252	559.35	0	100
Lactobacillus jensenii	UMB7383	EST50	Baseline	2017-04-20	urinary tract	urine (catheter)	SAMN38148632	73	41614	1733335	375.217	0	100
Lactobacillus jensenii	UMB7393	EST50	Baseline	2017-04-20	vagina	swab	SAMN38148633	66	50181	1745448	340.822	0	100
Lactobacillus paragasseri	UMB1891	EST07	Baseline	2016-01-21	urinary tract	urine (catheter)	SAMN38148618	39	99212	1983964	142.755	0	100
Lactobacillus paragasseri	UMB1985	EST07	Baseline	2016-01-21	urinary tract	urine (void)	SAMN38148613	37	99235	1982140	155.916	0	100
Lactobacillus paragasseri	UMB2003	EST07	Baseline	2016-01-21	vagina	swab	SAMN38148615	40	94935	1983625	206.09	0	100
Lactobacillus paragasseri	UMB2014	EST07	Baseline	2016-01-21	urinary tract	urine (catheter)	SAMN38148614	42	89055	1984399	188.388	0	100
Lactobacillus paragasseri	UMB2049	EST07	Baseline	2016-01-21	urinary tract	urine (catheter)	SAMN38148616	40	94969	1983775	227.133	0	100
Lactobacillus paragasseri	UMB2060	EST07	Baseline	2016-01-21	urinary tract	urine (catheter)	SAMN38148617	40	94969	1984371	172.352	0	100
Lactobacillus paragasseri	UMB3564	EST11	12-week	2016-05-23	vagina	swab	SAMN38148622	43	528966	1927781	262.372	0.3	100
Lactobacillus paragasseri	UMB3579	EST11	12-week	2016-05-23	urinary tract	urine (void)	SAMN38148623	21	528966	1897939	192.443	0.6	100
Lactobacillus paragasseri	UMB4933	EST20	12-week	2016-08-01	vagina	swab	SAMN38148651	18	334260	1951611	154.446	0.3	100
Lactobacillus paragasseri	UMB4951	EST20	12-week	2016-08-01	urinary tract	urine (void)	SAMN38148652	19	179872	1950987	241.397	0	100
Lactobacillus paragasseri	UMB4892	EST26	Baseline	2016-08-01	vagina	swab	SAMN38148625	9	1126262	1978148	142.567	0.3	100
Lactobacillus paragasseri	UMB4898	EST26	Baseline	2016-08-01	urinary tract	urine (catheter)	SAMN38148626	10	1124696	1975692	150.323	0.3	100
Lactobacillus paragasseri	UMB4908	EST26	Baseline	2016-08-01	urinary tract	urine (void)	SAMN38148624	12	1054408	1978961	152.778	0.3	100
Lactobacillus paragasseri	UMB6098	EST30	12-week	2016-12-15	urinary tract	urine (catheter)	SAMN38148648	20	260157	1976042	240.357	0	100
Lactobacillus paragasseri	UMB6101	EST30	12-week	2016-12-15	vagina	swab	SAMN38148647	19	260157	1975841	127.89	0	100
Lactobacillus paragasseri	UMB6107	EST30	12-week	2016-12-15	urinary tract	urine (void)	SAMN38148646	23	203430	2054252	169.287	0.3	100
Lactobacillus paragasseri	UMB7013	EST37	12-week	2017-03-23	urinary tract	urine (catheter)	SAMN38148649	14	237100	1959382	240.711	0.3	99.7
Lactobacillus paragasseri	UMB7023	EST37	12-week	2017-03-23	vagina	swab	SAMN38148650	53	79015	2078861	192.603	0.3	99.7

Supplementary Table 2. Average ANI, average pairwise identity, and %SNPs for each species' genome

**Species**	**Participant ID**	**Study Collection Timepoint**	**Average ANI**	**Average Pairwise Identity**
L. crispatus	EST21	Baseline	99.9938%	98.1%
L. crispatus	EST10	12-week	99.9855%	98.2%
L. crispatus	EST28	Baseline	99.9982%	98.3%
L. crispatus	EST28	12-week	99.9965%	98.2%
L. gasseri	EST43	Baseline	99.9986%	98.3%
L. gasseri	EST06	12-week	99.9990%	98.4%
L. iners	EST12	12-week	99.9995%	98.2%
L. iners	EST16	12-week	99.9976%	98.2%
L. jensenii	EST50	Baseline	99.9912%	98.3%
L. jensenii	EST17	Baseline	99.9782%	98.3%
L. jensenii	EST32	12-week	99.9941%	98.3%
L. paragasseri	EST11	12-week	99.9972%	98.4%
L. paragasseri	EST37	12-week	98.1906%	96.3%
L. paragasseri	EST07	Baseline	99.9980%	98.3%
L. paragasseri	EST26	Baseline	99.9966%	98.4%
L. paragasseri	EST30	12-week	98.9643%	97.3%
L. paragasseri	EST20	12-week	99.9912%	98.4%

s collected from the same participant and collection time.

SNPs (%)
0.1088
0.0767
0.0281
2.6277
0.0661
0.0208
0.1672
0.2440
0.0888
0.0517
0.1291
0.0745
2.1583
0.0307
0.0211
1.9883
0.0446

Supplementary Table 3. CRISPR analysis summary.

Species	Participant ID	Study Collection Timepoint	No. of Genomes Analyzed	No. of Genomes with CRISPR Arrays	No. of Different Arrays	Notes
L. crispatus	EST21	Baseline	2	2	1	
L. crispatus	EST10	Baseline	1	1	1	
L. crispatus	EST10	12-week	3	3	1	
L. crispatus	EST28	Baseline	2	2	1	
L. crispatus	EST28	12-week	3	3	1	
L. gasseri	EST43	Baseline	2	0	0	
L. gasseri	EST06	12-week	3	0	0	
L. iners	EST12	12-week	3	3	1	
L. iners	EST16	12-week	2	2	1	
L. jensenii	EST50	Baseline	2	2	1	
L. jensenii	EST17	Baseline	3	3	2	perineal=vaginal; void different
L. jensenii	EST32	12-week	2	2	2	void sample has 16 spacers; perineal has 17 spacers
L. paragasseri	EST11	12-week	2	2	1	
L. paragasseri	EST37	12-week	2	1	1	Cath sample doesn't have CRISPR
L. paragasseri	EST07	Baseline	6	6	1	
L. paragasseri	EST26	Baseline	3	0	0	
L. paragasseri	EST30	12-week	3	3	2	Cath = vaginal; void different
L. paragasseri	EST20	12-week	2	1	1	

Supplementary Table 4. Pairwise ANI values with empirical p based off of the simulated genome ANI distribution.

(A) *L. crispatus* comparisons. Rows highlighted in red are pairwise comparisons between strains collected during two different timepoints.

Participant ID/Collection Timepoint/Sample Type	Participant ID/Collection Timepoint/Sample Type	ANI	Empirical p
EST28 12-week cath	EST28 12-week void	99.9943	0.9121
EST28 12-week cath	EST28 12-week vaginal	99.9955	0.9331
EST28 12-week void	EST28 12-week vaginal	99.9996	1
EST28 Baseline void	EST28 Baseline vaginal	99.9982	0.98515
EST10 12-week void	EST10 12-week vaginal	99.9824	0.707671429
EST10 12-week void	EST10 12-week cath	99.9806	0.684766667
EST10 12-week cath	EST10 12-week vaginal	99.9935	0.89895
EST21 Baseline cath	EST21 Baseline vaginal	99.9938	0.9025
**Different Participants/Time**			
EST10 Baseline cath	EST28 12-week void	97.5917	0.0041
EST10 Baseline cath	EST28 12-week vaginal	97.4985	0.0041
EST10 Baseline cath	EST28 Baseline void	97.6414	0.0041
EST10 Baseline cath	EST28 Baseline vaginal	97.6004	0.0041
EST10 Baseline cath	EST10 12-week void	99.9808	0.68845
EST10 Baseline cath	EST10 12-week cath	99.9933	0.8937
EST10 Baseline cath	EST10 12-week vaginal	99.9735	0.61
EST10 Baseline cath	EST21 Baseline cath	98.5535	0.0041
EST10 Baseline cath	EST21 Baseline vaginal	99.0241	0.0041
EST28 12-week cath	EST28 Baseline void	99.9904	0.817125
EST28 12-week cath	EST28 Baseline vaginal	99.9981	0.9838
EST28 12-week cath	EST10 12-week void	97.5115	0.0041
EST28 12-week cath	EST10 12-week cath	97.3749	0.0041
EST28 12-week cath	EST10 12-week vaginal	97.4805	0.0041
EST28 12-week cath	EST21 Baseline cath	97.2213	0.0041
EST28 12-week cath	EST21 Baseline vaginal	97.3007	0.0041
EST28 12-week void	EST28 Baseline void	99.9929	0.8645
EST28 12-week void	EST28 Baseline vaginal	99.9952	0.927333333
EST28 12-week void	EST10 12-week void	97.6563	0.0041

EST28 12-week void	EST10 12-week cath	97.5199	0.0041
EST28 12-week void	EST10 12-week vaginal	97.5277	0.0041
EST28 12-week void	EST21 Baseline cath	97.3726	0.0041
EST28 12-week void	EST21 Baseline vaginal	97.4029	0.0041
EST28 12-week vaginal	EST28 Baseline void	99.9938	0.9025
EST28 12-week vaginal	EST28 Baseline vaginal	99.9953	0.928016667
EST28 12-week vaginal	EST10 12-week void	97.5416	0.0041
EST28 12-week vaginal	EST10 12-week cath	97.4982	0.0041
EST28 12-week vaginal	EST10 12-week vaginal	97.5145	0.0041
EST28 12-week vaginal	EST21 Baseline cath	97.1915	0.0041
EST28 12-week vaginal	EST21 Baseline vaginal	97.2631	0.0041
EST28 Baseline void	EST10 12-week void	97.5099	0.0041
EST28 Baseline void	EST10 12-week cath	97.3975	0.0041
EST28 Baseline void	EST10 12-week vaginal	97.4697	0.0041
EST28 Baseline void	EST21 Baseline cath	97.275	0.0041
EST28 Baseline void	EST21 Baseline vaginal	97.2902	0.0041
EST28 Baseline vaginal	EST10 12-week void	97.5697	0.0041
EST28 Baseline vaginal	EST10 12-week cath	97.5355	0.0041
EST28 Baseline vaginal	EST10 12-week vaginal	97.4767	0.0041
EST28 Baseline vaginal	EST21 Baseline cath	97.3123	0.0041
EST28 Baseline vaginal	EST21 Baseline vaginal	97.3731	0.0041
EST10 12-week void	EST21 Baseline cath	98.5881	0.0041
EST10 12-week void	EST21 Baseline vaginal	98.8402	0.0041
EST10 12-week cath	EST21 Baseline cath	98.9496	0.0041
EST10 12-week cath	EST21 Baseline vaginal	98.9629	0.0041
EST10 12-week vaginal	EST21 Baseline cath	98.6958	0.0041
EST10 12-week vaginal	EST21 Baseline vaginal	98.7223	0.0041

Supplementary Table 4. Pairwise ANI values with empirical p based off of the simulated genome ANI distribution.
(B) *L. gasseri* comparisons.

Participant ID/Collection Timepoint/Sample Type	Participant ID/Collection Timepoint/Sample Type	ANI	Empirical p
EST06 12-week void	EST06 12-week vaginal	99.9985	1
EST06 12-week void	EST 12-week cath	99.999	1
EST06 12-week vaginal	EST 12-week cath	99.9995	1
EST43 Baseline cath	EST43 Baseline vaginal	99.9986	1
**Different Participants/Time**			
EST06 12-week void	EST10 Baseline cath	98.9111	0.0094
EST06 12-week void	EST43 Baseline cath	98.9905	0.0094
EST06 12-week void	EST43 Baseline vaginal	98.9975	0.0094
EST06 12-week vaginal	EST10 Baseline cath	98.9637	0.0094
EST06 12-week vaginal	EST43 Baseline cath	99.0079	0.0094
EST06 12-week vaginal	EST43 Baseline vaginal	98.9878	0.0094
EST 12-week cath	EST10 Baseline cath	98.9593	0.0094
EST 12-week cath	EST43 Baseline cath	98.9797	0.0094
EST 12-week cath	EST43 Baseline vaginal	98.9657	0.0094
EST10 Baseline cath	EST43 Baseline cath	99.5201	0.0094
EST10 Baseline cath	EST43 Baseline vaginal	99.5168	0.0094

Supplementary Table 4. Pairwise ANI values with empirical p based off of the simulated genome ANI distribution.
(C) *L. iners* comparisons.

Participant ID/Collection Timepoint/Sample Type	Participant ID/Collection Timepoint/Sample Type	ANI	Empirical p
EST16 12-week vaginal	EST16 12-week cath	99.9995	0.9759
EST12 12-week void	EST12 12-week vaginal	99.997	0.5378
EST12 12-week void	EST12 12-week cath	99.9969	0.5302
EST12 12-week vaginal	EST12 12-week cath	99.9988	0.8097
**Different Participants/Time**			
EST16 12-week vaginal	EST12 12-week void	98.9685	0.0065
EST16 12-week vaginal	EST12 12-week vaginal	98.9445	0.0065
EST16 12-week vaginal	EST12 12-week cath	98.8916	0.0065
EST16 12-week cath	EST12 12-week void	98.9685	0.0065
EST16 12-week cath	EST12 12-week vaginal	98.9855	0.0065
EST16 12-week cath	EST12 12-week cath	98.9421	0.0065

Supplementary Table 4. Pairwise ANI values with empirical p based off of the simulated genome ANI distribution.
(D) *L. jensenii* comparisons. Rows highlighted in red are pairwise comparisons between strains collected during two different timepoints.

Participant ID/Collection Timepoint/Sample Type	Participant ID/Collection Timepoint/Sample Type	ANI	Empirical p
EST17 Baseline void	EST17 Baseline vaginal	99.9618	0.018725564
EST17 Baseline void	EST17 Baseline perineal	99.9816	0.069
EST17 Baseline vaginal	EST17 Baseline perineal	99.9913	0.256433333
EST50 Baseline cath	EST50 Baseline vaginal	99.9912	0.2549
EST32 12-week perineal	EST32 12-week void	99.9941	0.365
**Different Participants/Time**			
EST32 Baseline cath	EST17 Baseline void	99.9669	0.030136735
EST32 Baseline cath	EST17 Baseline vaginal	99.9417	0.0047
EST32 Baseline cath	EST17 Baseline perineal	99.9514	0.01497218
EST32 Baseline cath	EST50 Baseline cath	99.8684	0.0047
EST32 Baseline cath	EST50 Baseline vaginal	99.7233	0.0047
EST32 Baseline cath	EST32 12-week perineal	99.989	0.17425
EST32 Baseline cath	EST32 12-week void	99.9962	0.5238
EST17 Baseline void	EST50 Baseline cath	99.8333	0.0047
EST17 Baseline void	EST50 Baseline vaginal	99.8553	0.0047
EST17 Baseline vaginal	EST50 Baseline cath	99.688	0.0047
EST17 Baseline vaginal	EST50 Baseline vaginal	99.786	0.0047
EST17 Baseline perineal	EST50 Baseline cath	99.8243	0.0047
EST17 Baseline perineal	EST50 Baseline vaginal	99.8172	0.0047
EST17 Baseline void	EST32 12-week perineal	99.9676	0.030879592
EST17 Baseline void	EST32 12-week void	99.9462	0.007671429
EST17 Baseline vaginal	EST32 12-week perineal	99.9692	0.032577551
EST17 Baseline vaginal	EST32 12-week void	99.9444	0.006185714
EST17 Baseline perineal	EST32 12-week perineal	99.9351	0.0047
EST17 Baseline perineal	EST32 12-week void	99.9605	0.018256391
EST50 Baseline cath	EST32 12-week perineal	99.7924	0.0047
EST50 Baseline cath	EST32 12-week void	99.8541	0.0047
EST50 Baseline vaginal	EST32 12-week perineal	99.6888	0.0047

EST50 Baseline vaginal	EST32 12-week void	99.8728	0.0047

Supplementary Table 4. Pairwise ANI values with empirical p based off of the simulated genome ANI distribution.
(E) *L. paragasseri* comparisons.

Participant ID/Collection Timepoint/Sample Type	Participant ID/Collection Timepoint/Sample Type	ANI	Empirical p
EST26 Baseline void	EST26 Baseline vaginal	99.9946	0.91198
EST26 Baseline void	EST26 Baseline cath	99.9979	0.9629
EST26 Baseline vaginal	EST26 Baseline cath	99.9974	0.9426
EST07 Baseline void	EST07 Baseline cath 1	99.9976	0.95
EST07 Baseline void	EST07 Baseline cath 3	99.997	0.9405
EST07 Baseline void	EST07 Baseline cath 2	99.9976	0.95
EST07 Baseline void	EST07 Baseline vaginal	99.9983	0.9677
EST07 Baseline void	EST07 Baseline cath 4	99.9989	0.9892
EST07 Baseline cath 1	EST07 Baseline cath 3	99.9985	0.9773
EST07 Baseline cath 1	EST07 Baseline cath 2	99.9973	0.942
EST07 Baseline cath 1	EST07 Baseline vaginal	99.9984	0.9718
EST07 Baseline cath 1	EST07 Baseline cath 4	99.9981	0.9654
EST07 Baseline cath 3	EST07 Baseline cath 2	99.9976	0.95
EST07 Baseline cath 3	EST07 Baseline vaginal	99.9981	0.9654
EST07 Baseline cath 3	EST07 Baseline cath 4	99.9985	0.9773
EST07 Baseline cath 2	EST07 Baseline vaginal	99.9984	0.9718
EST07 Baseline cath 2	EST07 Baseline cath 4	99.9983	0.9677
EST07 Baseline vaginal	EST07 Baseline cath 4	99.9981	0.9654
EST30 12-week cath	EST30 12-week void	98.4588	0.002
EST30 12-week cath	EST30 12-week vaginal	99.9992	0.99915
EST30 12-week void	EST30 12-week vaginal	98.4349	0.002
EST11 12-week vaginal	EST11 12-week void	99.9972	0.9414
EST37 12-week vaginal	EST37 12-week cath	98.1906	0.002
EST20 12-week void	EST20 12-week vaginal	99.9912	0.88176
**Different Participants/Time**			
EST26 Baseline void	EST07 Baseline void	98.4495	0.002
EST26 Baseline void	EST07 Baseline cath 1	98.3991	0.002
EST26 Baseline void	EST07 Baseline cath 3	98.3802	0.002

EST26 Baseline void	EST07 Baseline cath 2	98.4364	0.002
EST26 Baseline void	EST07 Baseline vaginal	98.4793	0.002
EST26 Baseline void	EST07 Baseline cath 4	98.4849	0.002
EST26 Baseline void	EST30 12-week cath	98.7995	0.002
EST26 Baseline void	EST30 12-week void	98.4198	0.002
EST26 Baseline void	EST30 12-week vaginal	98.7688	0.002
EST26 Baseline void	EST11 12-week vaginal	98.2694	0.002
EST26 Baseline void	EST11 12-week void	98.2969	0.002
EST26 Baseline void	EST37 12-week vaginal	98.3895	0.002
EST26 Baseline void	EST37 12-week cath	98.7208	0.002
EST26 Baseline void	EST20 12-week void	98.8504	0.002
EST26 Baseline void	EST20 12-week vaginal	98.8997	0.002
EST26 Baseline vaginal	EST07 Baseline void	98.3875	0.002
EST26 Baseline vaginal	EST07 Baseline cath 1	98.3668	0.002
EST26 Baseline vaginal	EST07 Baseline cath 3	98.3553	0.002
EST26 Baseline vaginal	EST07 Baseline cath 2	98.3741	0.002
EST26 Baseline vaginal	EST07 Baseline vaginal	98.4545	0.002
EST26 Baseline vaginal	EST07 Baseline cath 4	98.3001	0.002
EST26 Baseline vaginal	EST30 12-week cath	98.8457	0.002
EST26 Baseline vaginal	EST30 12-week void	98.4041	0.002
EST26 Baseline vaginal	EST30 12-week vaginal	98.8116	0.002
EST26 Baseline vaginal	EST11 12-week vaginal	98.1935	0.002
EST26 Baseline vaginal	EST11 12-week void	98.207	0.002
EST26 Baseline vaginal	EST37 12-week vaginal	98.2858	0.002
EST26 Baseline vaginal	EST37 12-week cath	98.673	0.002
EST26 Baseline vaginal	EST20 12-week void	98.9265	0.002
EST26 Baseline vaginal	EST20 12-week vaginal	98.9	0.002
EST26 Baseline cath	EST07 Baseline void	98.3741	0.002
EST26 Baseline cath	EST07 Baseline cath 1	98.4633	0.002
EST26 Baseline cath	EST07 Baseline cath 3	98.354	0.002
EST26 Baseline cath	EST07 Baseline cath 2	98.4099	0.002
EST26 Baseline cath	EST07 Baseline vaginal	98.4688	0.002
EST26 Baseline cath	EST07 Baseline cath 4	98.2637	0.002

EST26 Baseline cath	EST30 12-week cath	98.8605	0.002
EST26 Baseline cath	EST30 12-week void	98.4418	0.002
EST26 Baseline cath	EST30 12-week vaginal	98.7453	0.002
EST26 Baseline cath	EST11 12-week vaginal	98.2217	0.002
EST26 Baseline cath	EST11 12-week void	98.2201	0.002
EST26 Baseline cath	EST37 12-week vaginal	98.2503	0.002
EST26 Baseline cath	EST37 12-week cath	98.6617	0.002
EST26 Baseline cath	EST20 12-week void	98.944	0.002
EST26 Baseline cath	EST20 12-week vaginal	98.9203	0.002
EST07 Baseline void	EST30 12-week cath	98.2617	0.002
EST07 Baseline void	EST30 12-week void	98.3377	0.002
EST07 Baseline void	EST30 12-week vaginal	98.2248	0.002
EST07 Baseline void	EST11 12-week vaginal	98.488	0.002
EST07 Baseline void	EST11 12-week void	98.2063	0.002
EST07 Baseline void	EST37 12-week vaginal	99.7229	0.1001
EST07 Baseline void	EST37 12-week cath	98.331	0.002
EST07 Baseline void	EST20 12-week void	98.3473	0.002
EST07 Baseline void	EST20 12-week vaginal	98.3311	0.002
EST07 Baseline cath 1	EST30 12-week cath	98.2458	0.002
EST07 Baseline cath 1	EST30 12-week void	98.3037	0.002
EST07 Baseline cath 1	EST30 12-week vaginal	98.1989	0.002
EST07 Baseline cath 1	EST11 12-week vaginal	98.4349	0.002
EST07 Baseline cath 1	EST11 12-week void	98.2254	0.002
EST07 Baseline cath 1	EST37 12-week vaginal	99.7182	0.0913
EST07 Baseline cath 1	EST37 12-week cath	98.3073	0.002
EST07 Baseline cath 1	EST20 12-week void	98.2902	0.002
EST07 Baseline cath 1	EST20 12-week vaginal	98.3135	0.002
EST07 Baseline cath 3	EST30 12-week cath	98.2722	0.002
EST07 Baseline cath 3	EST30 12-week void	98.3618	0.002
EST07 Baseline cath 3	EST30 12-week vaginal	98.1978	0.002
EST07 Baseline cath 3	EST11 12-week vaginal	98.3292	0.002
EST07 Baseline cath 3	EST11 12-week void	98.1365	0.002
EST07 Baseline cath 3	EST37 12-week vaginal	99.7259	0.10187551

EST07 Baseline cath 3	EST37 12-week cath	98.3238	0.002
EST07 Baseline cath 3	EST20 12-week void	98.3913	0.002
EST07 Baseline cath 3	EST20 12-week vaginal	98.3573	0.002
EST07 Baseline cath 2	EST30 12-week cath	98.2559	0.002
EST07 Baseline cath 2	EST30 12-week void	98.3221	0.002
EST07 Baseline cath 2	EST30 12-week vaginal	98.2281	0.002
EST07 Baseline cath 2	EST11 12-week vaginal	98.3547	0.002
EST07 Baseline cath 2	EST11 12-week void	98.1777	0.002
EST07 Baseline cath 2	EST37 12-week vaginal	99.713	0.088886957
EST07 Baseline cath 2	EST37 12-week cath	98.3599	0.002
EST07 Baseline cath 2	EST20 12-week void	98.3518	0.002
EST07 Baseline cath 2	EST20 12-week vaginal	98.2933	0.002
EST07 Baseline vaginal	EST30 12-week cath	98.3153	0.002
EST07 Baseline vaginal	EST30 12-week void	98.4917	0.002
EST07 Baseline vaginal	EST30 12-week vaginal	98.327	0.002
EST07 Baseline vaginal	EST11 12-week vaginal	98.3819	0.002
EST07 Baseline vaginal	EST11 12-week void	98.4825	0.002
EST07 Baseline vaginal	EST37 12-week vaginal	99.7002	0.08292449
EST07 Baseline vaginal	EST37 12-week cath	98.2591	0.002
EST07 Baseline vaginal	EST20 12-week void	98.3543	0.002
EST07 Baseline vaginal	EST20 12-week vaginal	98.2992	0.002
EST07 Baseline cath 4	EST30 12-week cath	98.323	0.002
EST07 Baseline cath 4	EST30 12-week void	98.3307	0.002
EST07 Baseline cath 4	EST30 12-week vaginal	98.1832	0.002
EST07 Baseline cath 4	EST11 12-week vaginal	98.3201	0.002
EST07 Baseline cath 4	EST11 12-week void	98.2012	0.002
EST07 Baseline cath 4	EST37 12-week vaginal	99.7286	0.103512821
EST07 Baseline cath 4	EST37 12-week cath	98.3128	0.002
EST07 Baseline cath 4	EST20 12-week void	98.3491	0.002
EST07 Baseline cath 4	EST20 12-week vaginal	98.3239	0.002
EST30 12-week cath	EST11 12-week vaginal	98.2908	0.002
EST30 12-week cath	EST11 12-week void	98.3802	0.002
EST30 12-week cath	EST37 12-week vaginal	98.0315	0.002

EST30 12-week cath	EST37 12-week cath	98.6387	0.002
EST30 12-week cath	EST20 12-week void	99.6999	0.082777551
EST30 12-week cath	EST20 12-week vaginal	99.7209	0.0931
EST30 12-week void	EST11 12-week vaginal	98.2588	0.002
EST30 12-week void	EST11 12-week void	98.2647	0.002
EST30 12-week void	EST37 12-week vaginal	98.3774	0.002
EST30 12-week void	EST37 12-week cath	98.4358	0.002
EST30 12-week void	EST20 12-week void	98.5056	0.002
EST30 12-week void	EST20 12-week vaginal	98.4613	0.002
EST30 12-week vaginal	EST11 12-week vaginal	98.1502	0.002
EST30 12-week vaginal	EST11 12-week void	98.1983	0.002
EST30 12-week vaginal	EST37 12-week vaginal	98.1087	0.002
EST30 12-week vaginal	EST37 12-week cath	98.5996	0.002
EST30 12-week vaginal	EST20 12-week void	99.7221	0.096433333
EST30 12-week vaginal	EST20 12-week vaginal	99.7119	0.088683696
EST11 12-week vaginal	EST37 12-week vaginal	98.3831	0.002
EST11 12-week vaginal	EST37 12-week cath	98.2062	0.002
EST11 12-week vaginal	EST20 12-week void	98.0866	0.002
EST11 12-week vaginal	EST20 12-week vaginal	98.1331	0.002
EST11 12-week void	EST37 12-week vaginal	98.4137	0.002
EST11 12-week void	EST37 12-week cath	98.2235	0.002
EST11 12-week void	EST20 12-week void	98.1328	0.002
EST11 12-week void	EST20 12-week vaginal	98.1574	0.002
EST37 12-week vaginal	EST20 12-week void	98.2531	0.002
EST37 12-week vaginal	EST20 12-week vaginal	98.3849	0.002
EST37 12-week cath	EST20 12-week void	98.4692	0.002
EST37 12-week cath	EST20 12-week vaginal	98.5076	0.002

Supplementary File 1. CRISPR spacer sequences identified for the Lactobacillus genomes.

L. jensenii UMB5669 | EST32 Baseline cath
ACTAACCTTAAATATTCATCGACTACTTTT
CTCGCTATTGAGTTGCGACTTCAAGTTTTC
ATCTTTTCTGCAACTGCCCATTCTGGTAGT
TTAGAAACATTAATCTTATTGTCTTTAGCC
TAGCTCATCGGCAATTTCCTGCAAAAATTT
AAGAAGCTTGACAGAGAAATTGAAAGTAGT
ACACTAAAAATGATGCTGAAAAAGCTTTAG
AGCCATCTAATGCTTCAGGCTTGATATATT
TTGTACAATATCCTTATTACGTATTGTCTT
TTACATGTATGTATGCAGATTAAAATAGAG
GCTCAATAGTCTTGTTTACTACTTTAAACA
AACTGATAAATGGAAGGCCTGCCAGTATCC
CCTTCTTGGCTCGATAAAACAGCTCAAAAA
CAGATGGATCCTATTCACTTGCTGATTCAA
TCTTCTCGCAATTCTTCTTGCATTTCGTCA
GTTTCATAGTCAATTTCATTGCGAAATTTA
TGACAATTAAGTTGGATAACGAAATCACAA

L. jensenii UMB3466 | EST17 Baseline perineal
TGAGTAATCGTGCTAGTGACAGAGCTAGC
TGTGATTTCGTTATCCAACTTAATTGTCA
AAATTTCGCAATGAAATTGACTATGAAAC
GACGAAATGCAAGAAGAATTGCGAGAAGA
TGAATCAGCAAGTGAATAGGATCCATCTG
TTTTGAGCTGTTTTATCGAGCCAAGAAGG
AGACAATACGTAATAAGGATATTGTACAA
ATATATCAAGCCTGAAGCATTAGATGGCT
TAAAGCTTTTTCAGCATCATTTTTAGTGT
CTACTTTCAATTTCTCTGTCAAGCTTCTT
AATTTTTGCAGGAAATTGCCGATGAGCTA
GCTAAAGACAATAAGATTAATGTTTCTAA
AAAGTAGTCGATGAATATTTAAGGTTAGT

L. jensenii UMB3451 | EST17 Baseline vaginal
ACTAACCTTAAATATTCATCGACTACTTT
TTAGAAACATTAATCTTATTGTCTTTAGC
TAGCTCATCGGCAATTTCCTGCAAAAATT
AAGAAGCTTGACAGAGAAATTGAAAGTAG
ACACTAAAAATGATGCTGAAAAAGCTTTA
AGCCATCTAATGCTTCAGGCTTGATATAT
TTGTACAATATCCTTATTACGTATTGTCT
CCTTCTTGGCTCGATAAAACAGCTCAAAA
CAGATGGATCCTATTCACTTGCTGATTCA
TCTTCTCGCAATTCTTCTTGCATTTCGTC
GTTTCATAGTCAATTTCATTGCGAAATTT
TGACAATTAAGTTGGATAACGAAATCACA

GCTAGCTCTGTCACTAGCACGATTACTCA

L. jensenii UMB3478 | EST17 Baseline void
TAACTTTTGAAGAATTAGTCATGAATATAA
TTGAGTAATCGTGCTAGTGACAGAGCTAGC
TTGTGATTTCGTTATCCAACTTAATTGTCA
TAAATTTCGCAATGAAATTGACTATGAAAC
TGACGAAATGCAAGAAGAATTGCGAGAAGA
TTGAATCAGCAAGTGAATAGGATCCATCTG
GGATACTGGCAGGCCTTCCATTTATCAGTT
AAGACAATACGTAATAAGGATATTGTACAA
AATATATCAAGCCTGAAGCATTAGATGGCT
CTAAAGCTTTTTCAGCATCATTTTTAGTGT
ACTACTTTCAATTTCTCTGTCAAGCTTCTT
AAATTTTTGCAGGAAATTGCCGATGAGCTA
GGCTAAAGACAATAAGATTAATGTTTCTAA
ACTACCAGAATGGGCAGTTGCAGAAAAGAT
GAAAACTTGAAGTCGCAACTCAATAGCGAG
AAAAGTAGTCGATGAATATTTAAGGTTAGT

L. jensenii UMB7383 | EST50 Baseline cath
ATTGAAAATCCAATACAAGCTTGCTGGAACA
CGATAGTGTCATCGTAAACAATAAATCCTT
TGAAGATGGAGAGATTGCTGCAGTTCTTGT
AAGCAAAAAAAGTATATCCAAAAGGAACGG
AAACGTGATTGCTGAAATTACTACAACCTT
GCTAGCGTGCTAGTAGTGATTACTGAGAGT
TTCCAATGGTGAAACAAGAGTTATCAAATA
TGTTGCAAGCGCCGACCCTTATGGTGGTAT
AAGACAATACGTAATAAGGATATTGCACAA
AATATATCAAGCCTGAAGCATTAGATGGCT
ACTACTTTCAATTTCTCTGTCAAGCTTCTT
TACGCCTAGTGGCTATATTGACTTAACTAG

L. jensenii UMB6506 | EST32 12-week perineal
TTGTGATTTCGTTATCCAACTTAATTGTCA
TAAATTTCGCAATGAAATTGACTATGAAAC
TGACGAAATGCAAGAAGAATTGCGAGAAGA
TTGAATCAGCAAGTGAATAGGATCCATCTG
TTTTTGAGCTGTTTTATCGAGCCAAGAAGG
GGATACTGGCAGGCCTTCCATTTATCAGTT
TGTTTAAAGTAGTAAACAAGACTATTGAGC
CTCTATTTTAATCTGCATACATACATGTAA
AAGACAATACGTAATAAGGATATTGTACAA
AATATATCAAGCCTGAAGCATTAGATGGCT
CTAAAGCTTTTTCAGCATCATTTTTAGTGT
ACTACTTTCAATTTCTCTGTCAAGCTTCTT
AAATTTTTGCAGGAAATTGCCGATGAGCTA
GGCTAAAGACAATAAGATTAATGTTTCTAA
ACTACCAGAATGGGCAGTTGCAGAAAAGAT

GAAAACTTGAAGTCGCAACTCAATAGCGAG
AAAAGTAGTCGATGAATATTTAAGGTTAGT

L. jensenii UMB7393 | EST50 Baseline vaginal
CTAGTTAAGTCAATATAGCCACTAGGCGTA
AAGAAGCTTGACAGAGAAATTGAAAGTAGT
AGCCATCTAATGCTTCAGGCTTGATATATT
TTGTGCAATATCCTTATTACGTATTGTCTT
ATACCACCATAAGGGTCGGCGCTTGCAACA
TATTTGATAACTCTTGTTTCACCATTGGAA
ACTCTCAGTAATCACTACTAGCACGCTAGC
AAGGTTGTAGTAATTTCAGCAATCACGTTT
CCGTTCCTTTTGGATATACTTTTTTTGCTT
ACAAGAACTGCAGCAATCTCTCCATCTTCA
AAGGATTTATTGTTTACGATGACACTATCG
TGTTCCAGCAAGCTTGTATTGGATTTTCAAT

L. jensenii UMB6491 | EST32 12-week void
CTCGCTATTGAGTTGCGACTTCAAGTTTTC
ATCTTTTCTGCAACTGCCCATTCTGGTAGT
TTAGAAACATTAATCTTATTGTCTTTAGCC
TAGCTCATCGGCAATTTCCTGCAAAAATTT
AAGAAGCTTGACAGAGAAATTGAAAGTAGT
ACACTAAAAATGATGCTGAAAAAGCTTTAG
AGCCATCTAATGCTTCAGGCTTGATATATT
TTGTACAATATCCTTATTACGTATTGTCTT
TTACATGTATGTATGCAGATTAAAATAGAG
GCTCAATAGTCTTGTTTACTACTTTAAACA
AACTGATAAATGGAAGGCCTGCCAGTATCC
CCTTCTTGGCTCGATAAAACAGCTCAAAAA
CAGATGGATCCTATTCACTTGCTGATTCAA
TCTTCTCGCAATTCTTCTTGCATTTCGTCA
GTTTCATAGTCAATTTCATTGCGAAATTTA
TGACAATTAAGTTGGATAACGAAATCACAA

L. paragasseri UMB4908 | EST26 Baseline void
No CRISPR

L. paragasseri UMB1985 | EST07 Baseline void
CGCCTTTTTGTCCGCCTTCTTTGATTAACT
TGTTGTCGCTGATTTCATTAATTATGCAAA
TATTCAGCATTACAACTCGTACCGACGATA
CGTGATTGACATATTGATGACCAGCAAACC
TGATCTGGGCGTAAATAAACGTCAGTAGCT
AGAAAATAAACTATTCCTGTTGCATTAATG
TTCTTGGCAGAGTTCTTTTTTAAAAAATAC
TATCTAAGCAAGATATTGAAAATAAGGCGA
TATTTTTGATAGTTCTTTTTTTAGTTTTAC
CAAAAATTTTGTTCATAATTCTACGTTCGT
CGCTGAATATACTTCTGATACGACTGTAAG

ACAGGCATTGGTATTTTTTGATAAACGCCA
CGCTAGTTAAAGAAAAAGGCTGGCCGATTA
ATGTACTAGTTATTTGCAACTTGGAAGACA
TAATCTCCTTATTTTGTGTAAACAAAAACA
TGCCTTACGCTTCTTTTCTTTGACTTCAAC

L. paragasseri UMB3564 | EST11 12-week vaginal
ATCCCAATTGGGATGCGTTTTTCAATGTTC
AATTGGTATCAAGAGGCGTATTCGAGTGGA
ATCGGCATGCTTTCTCAAATATGGCATCAC
CCACTTGCTGCCGTTGTAGTAGTCGTTGGA
GATACTACGACGAAGTACAGCAAGAGTTAAA
AGTACATCACACTGCTCGACCCGCTCACCG
TTAAACGGTTAGAAAACAAGATAGATGATT
TCTACCATGGCAAGAAGCTCCGCGTGCCCG
AACTCACTGCGTTGAGAGCACTGCTCTGCA
GCTAAGGCAATCCCTTGCTGCTGGCGCTCAAT
TTAGGTAATGAACCAGATGAAGCAAATTAC
CCAGTTATTGGAAGTTATGCCGGTTATGGT
TTTTTACCATGAAACTACGGATAAAATACA
TTTCTAGATCTAGATTAGTGTTGTCTTGTG
TATCTTTTAACTTAACAATATTTTCATAGT
TGTTGATCTCGATAATACTCCCATTCGCTG
AAGACTTCTTGGTATCCTTGGTCCATTTTT
GTATCTCCGCCACTTGCTACTTTTTTGTCA
AACAAGCGCCGGGATTAGGTGCGGCTTTGT

L. gasseri UMB3290 | EST06 12-week void
No CRISPR

L. gasseri UMB3277 | EST06 12-week vaginal
No CRISPR

L. paragasseri UMB3579 | EST11 12-week void
ATCCCAATTGGGATGCGTTTTTCAATGTTC
AATTGGTATCAAGAGGCGTATTCGAGTGGA
ATCGGCATGCTTTCTCAAATATGGCATCAC
CCACTTGCTGCCGTTGTAGTAGTCGTTGGA
GATACTACGACGAAGTACAGCAAGAGTTAAA
AGTACATCACACTGCTCGACCCGCTCACCG
TTAAACGGTTAGAAAACAAGATAGATGATT
TCTACCATGGCAAGAAGCTCCGCGTGCCCG
AACTCACTGCGTTGAGAGCACTGCTCTGCA
GCTAAGGCAATCCCTTGCTGCTGGCGCTCAAT
TTAGGTAATGAACCAGATGAAGCAAATTAC
CCAGTTATTGGAAGTTATGCCGGTTATGGT
TTTTTACCATGAAACTACGGATAAAATACA
TTTCTAGATCTAGATTAGTGTTGTCTTGTG
TATCTTTTAACTTAACAATATTTTCATAGT
TGTTGATCTCGATAATACTCCCATTCGCTG

AAGACTTCTTGGTATCCTTGGTCCATTTTT
GTATCTCCGCCACTTGCTACTTTTTTGTCA
AACAAGCGCCGGGATTAGGTGCGGCTTTGT

L. gasseri UMB3275 | EST06 12-week cath
No CRISPR

L. paragasseri UMB2014 | EST07 Baseline cath1
GTTGAAGTCAAAGAAAAGAAGCGTAAGGCA
TGTTTTTGTTTACACAAAATAAGGAGATTA
TGTCTTCCAAGTTGCAAATAACTAGTACAT
TAATCGGCCAGCCTTTTTCTTTAACTAGCG
TGGCGTTTATCAAAAAATACCAATGCCTGT
CTTACAGTCGTATCAGAAGTATATTCAGCG
ACGAACGTAGAATTATGAACAAAATTTTTG
GTAAAACTAAAAAAAGAACTATCAAAAATA
TCGCCTTATTTTCAATATCTTGCTTAGATA
GTATTTTTTAAAAAAGAACTCTGCCAAGAA
CATTAATGCAACAGGAATAGTTTATTTTCT
AGCTACTGACGTTTATTTACGCCCAGATCA
GGTTTGCTGGTCATCAATATGTCAATCACG
TATCGTCGGTACGAGTTGTAATGCTGAATA
TTTGCATAATTAATGAAATCAGCGACAACA
AGTTAATCAAAGAAGGCGGACAAAAAGGCG

L. paragasseri UMB7023 | EST37 12-week vaginal
TTAAATGGCACAAAAATAGCTGGATATAGG
AATTTTAAAATTGGTCATGTATAATTACAC
ATTTATCAAATGGATTAATCAGCAAGTGGA
TTTAGAAGTTAATGGTACACCTTGGTTCGT
TTTAGCTAGTGTGCTTGGCTATTCTAATAC
TTTTAAGTCAAGAATTTCGGCGTCATCAAA
TGAAGCAACGTCCAGAGACCAATATAGCCG
TTTGATGGTTGGCGCCGCAATGGTGATCCC
AATGGCACTTGACCATTGTTTTCGGTGATC
CTGGTAGTGAGAGTACCAGTCATAGCAAGG
CAGAACTAAAGCTAGTTTCACCTTCTTATC
ACAAAAACGAATTACCATTCATTACAGCGC
TAGGCGAACAATGTATCACTGGTCCTACTT
CTCAGCTGCATCATTTAGTGCAACTTTAAG
CATCAGGGACACAAATTCAAACTTATAAAA
GTAGGAGGTAACATATATGGAAGTAGCAAA
TGTCTTCCAAGTTGCAAATAACTAGTACAT
TACGATGATGTTCTTTTAACAATTGAAACA
AGTTGGGTAAAAGACAAAGTTGACGATTTA
ATCGAACCTAAATGCTGCTGTACATCAATC
GCATCTACAATAAAAATATGCCCGATTCCA
TTTCTCCAATGGAACCAAGTCGATTTGGGT
TATCGCATTATTCATCGTATCCATACCCGT
TATCGTCGGTACGAGTTGTAATGCTGAATA

TTTGCATAATTAATGAAATCAGCGACAACA
AGTTAATCAAAGAAGGCGGACAAAAAGGCG

L. crispatus UMB2389 | EST10 Baseline cath
AGAAAACAGGTATTCCAGGCGCGATAGTGG
TACCTCACACCTCTATTATTTATTTCTTTAGATCTAAACCTCATTGATCTAAAACCGTACGTTGGTACCG
CCGCAAAAGCTAATA
GTCTTACGCATGTAAACGCCGTTTTCACGG
CCGCGTTAATGTTGATTTTTCTAAATTCGA
GTTCCTAGCAGGGACAAGATTCAAGTGCTT
ACGTAAGCACCTTTTGAATTGGAGACTACT
TAAGCCTCATCTTTTGGCTGATGTAACGACTTGGATCA
AAACGGCTGTGGCGTTGACACAGCTCAAAGTAGGATCA
CTAACTGGATACCAGTCATCACCGTTAACAGTAGGATCA
CTGCCTTGTCTCCCGTGCCCTTCAAGATGATAGGATCA

L. crispatus UMB6018 | EST28 12-week cath
CAGTTTAGGTACCATTTTTTGACGATCAAAATC
TGACAAGGTTGACCAAGCCGTTAAGACCATGCAA
TTAAAATAATTAAGGAGGGAACTCCAAATATCG
CAATTTCTTCCTTCGACATGTCCTTAAGAGCAT
TGACAGCTCTGCTGTAGTGATCGAATCAGACAA
TCCGACGGGGTACCCCGCGGCTGTCCATCATC
CGTGTAATGAATACGTTTAGCGTACGTATTCCG
CCAAATCTTCTAAAAATGCAATAGATATTTAGA
CAATGAAATTTTTAATGGCTGATATATTGAGTG
TGTTTCTCTAGCTGGTGGTACCAATGGTACTAA
TAAAAAAGTAATTGGTGATCCTAAAACGTATTC
TAAACGCACAGGCGTATTAGTGGGCGGACACCA
CATGTGGGCAAGGTTCAATTCCTTGCTAGTTCT
CATGGTTTCTCATTGCATCATTATCATCAACTA
TCGCTTGGTTACGGGCTAGGCAAAGCAAGCTTT
CTTGATAGCCTTGTACTTCTTTAATTGATCCAT
CACTATAACACAAAAAAAAGCCACTCCAGAGAA
TTACCCAAACCTGGAAGAGAACCCGTGTAGAAG
TCATCACGTATACTTTCACGATTGATGACAATA
CGTGTTGTTTCCATATTCATTAGATAAAACATC
TCTCTTGAAGACCATCTTTTTGGTCCTCAGTCA
TAACAATATGACCGCTACTGAATTGTTGAAGCA
TTAATGGCTGATGCAACAGCAGTCACACCATTT
CGTATAAAAAGCTAATTTGTACGCGTTTTGTTT
CATATCATGTGTTGACCAACCTAAATTTTCCAG
TAGTATTTTTTCTGTGCCTCTCTCAAGCACATT
TAAGCAAAAAGCTTTAAATATTTTCAATCAAGCT
TGGAATCATCAGAAACTAAATCAAAGAATTTTA
TACGTTTTAGGACATTCTAGAGTGACAACTGCA
CGCTTGCAGAATCCATTGAAGTCTCAATATCTT
CCATTGTCCCCGAACCAGCCTGCCGTACTGTGC
TGATTTCTTTAACCGCTTTTTGCAAAATGTCAT
ATTATTAGCAAGCTATTTATTTTCATGGAGGAG

TAGCATTGGTGCAGCAGGTTTATGGCAATTTTA
CCTACAATGATGCAAGAATGAGTAGCATGAAAG
CACTGTAATTGAAGATACAAAACTATCGTGGAA
TATGCAGAGTGGCGTGCATGTCATGATTACAGG

L. paragasseri UMB4951 | EST20 12-week void
No CRISPR

L. paragasseri UMB2060 | EST07 Baseline cath3
CGCCTTTTTGTCCGCCTTCTTTGATTAACT
TGTTGTCGCTGATTTCATTAATTATGCAAA
TATTCAGCATTACAACTCGTACCGACGATA
CGTGATTGACATATTGATGACCAGCAAACC
TGATCTGGGCGTAAATAAACGTCAGTAGCT
AGAAAATAAACTATTCCTGTTGCATTAATG
TTCTTGGCAGAGTTCTTTTTTAAAAAATAC
TATCTAAGCAAGATATTGAAAATAAGGCGA
TATTTTTGATAGTTCTTTTTTTAGTTTTAC
CAAAAATTTTGTTCATAATTCTACGTTCGT
CGCTGAATATACTTCTGATACGACTGTAAG
ACAGGCATTGGTATTTTTTGATAAACGCCA
CGCTAGTTAAAGAAAAAGGCTGGCCGATTA
ATGTACTAGTTATTTGCAACTTGGAAGACA
TAATCTCCTTATTTTGTGTAAACAAAAACA
TGCCTTACGCTTCTTTTCTTTGACTTCAAC

L. crispatus UMB5393 | EST28 Baseline void
CAGTTTAGGTACCATTTTTTGACGATCAAAATC
TGACAAGGTTGACCAAGCCGTTAAGACCATGCAA
TTAAAATAATTAAGGAGGGAACTCCAAATATCG
CAATTTCTTCCTTCGACATGTCCTTAAGAGCAT
TGACAGCTCTGCTGTAGTGATCGAATCAGACAA
TCCGACGGGGTACCCCGCGGCTGTCCATCATC
CGTGTAATGAATACGTTTAGCGTACGTATTCCG
CCAAATCTTCTAAAAATGCAATAGATATTTAGA
CAATGAAATTTTTAATGGCTGATATATTGAGTG
TGTTTCTCTAGCTGGTGGTACCAATGGTACTAA
TAAAAAAGTAATTGGTGATCCTAAAACGTATTC
TAAACGCACAGGCGTATTAGTGGGCGGACACCA
CATGTGGGCAAGGTTCAATTCCTTGCTAGTTCT
CATGGTTTCTCATTGCATCATTATCATCAACTA
TCGCTTGGTTACGGGCTAGGCAAAGCAAGCTTT
CTTGATAGCCTTGTACTTCTTTAATTGATCCAT
CACTATAACACAAAAAAAAAGCCACTCCAGAGAA
TTACCCAAACCTGGAAGAGAACCCGTGTAGAAG
TCATCACGTATACTTTCACGATTGATGACAATA
CGTGTTGTTTCCATATTCATTAGATAAAACATC
TCTCTTGAAGACCATCTTTTTGGTCCTCAGTCA
TAACAATATGACCGCTACTGAATTGTTGAAGCA
TTAATGGCTGATGCAACAGCAGTCACACCATTT

CGTATAAAAAGCTAATTTGTACGCGTTTTGTTT
CATATCATGTGTTGACCAACCTAAATTTTCCAG
TAGTATTTTTTCTGTGCCTCTCTCAAGCACATT
TAAGCAAAAAGCTTTAAATATTTTCAATCAAGCT
TGGAATCATCAGAAACTAAATCAAAGAATTTTA
TACGTTTTAGGACATTCTAGAGTGACAACTGCA
CGCTTGCAGAATCCATTGAAGTCTCAATATCTT
CCATTGTCCCCGAACCAGCCTGCCGTACTGTGC
TGATTTCTTTAACCGCTTTTTGCAAAATGTCAT
ATTATTAGCAAGCTATTTATTTTCATGGAGGAG
TAGCATTGGTGCAGCAGGTTTATGGCAATTTTA
CCTACAATGATGCAAGAATGAGTAGCATGAAAG
CACTGTAATTGAAGATACAAAACTATCGTGGAA
TATGCAGAGTGGCGTGCATGTCATGATTACAGG

L. paragasseri UMB2049 | EST07 Baseline cath2
CGCCTTTTTGTCCGCCTTCTTTGATTAACT
TGTTGTCGCTGATTTCATTAATTATGCAAA
TATTCAGCATTACAACTCGTACCGACGATA
CGTGATTGACATATTGATGACCAGCAAACC
TGATCTGGGCGTAAATAAACGTCAGTAGCT
AGAAAATAAACTATTCCTGTTGCATTAATG
TTCTTGGCAGAGTTCTTTTTTAAAAAATAC
TATCTAAGCAAGATATTGAAAATAAGGCGA
TATTTTTGATAGTTCTTTTTTTAGTTTTAC
CAAAAATTTTGTTCATAATTCTACGTTCGT
CGCTGAATATACTTCTGATACGACTGTAAG
ACAGGCATTGGTATTTTTTGATAAACGCCA
CGCTAGTTAAAGAAAAAGGCTGGCCGATTA
ATGTACTAGTTATTTGCAACTTGGAAGACA
TAATCTCCTTATTTTGTGTAAACAAAAACA
TGCCTTACGCTTCTTTTCTTTGACTTCAAC

L. paragasseri UMB2003 | EST07 Baseline vaginal
GTTGAAGTCAAAGAAAAGAAGCGTAAGGCA
TGTTTTTGTTTACACAAAATAAGGAGATTA
TGTCTTCCAAGTTGCAAATAACTAGTACAT
TAATCGGCCAGCCTTTTTCTTTAACTAGCG
TGGCGTTTATCAAAAAATACCAATGCCTGT
CTTACAGTCGTATCAGAAGTATATTCAGCG
ACGAACGTAGAATTATGAACAAAATTTTTG
GTAAAACTAAAAAAAGAACTATCAAAAATA
TCGCCTTATTTTCAATATCTTGCTTAGATA
GTATTTTTTAAAAAAGAACTCTGCCAAGAA
CATTAATGCAACAGGAATAGTTTATTTTCT
AGCTACTGACGTTTATTTACGCCCAGATCA
GGTTTGCTGGTCATCAATATGTCAATCACG
TATCGTCGGTACGAGTTGTAATGCTGAATA
TTTGCATAATTAATGAAATCAGCGACAACA
AGTTAATCAAAGAAGGCGGACAAAAAGGCG

L. paragasseri UMB1891 | EST07 Baseline cath4
CGCCTTTTTGTCCGCCTTCTTTGATTAACT
TGTTGTCGCTGATTTCATTAATTATGCAAA
TATTCAGCATTACAACTCGTACCGACGATA
CGTGATTGACATATTGATGACCAGCAAACC
TGATCTGGGCGTAAATAAACGTCAGTAGCT
AGAAAATAAACTATTCCTGTTGCATTAATG
TTCTTGGCAGAGTTCTTTTTTAAAAAATAC
TATCTAAGCAAGATATTGAAAATAAGGCGA
TATTTTTGATAGTTCTTTTTTTAGTTTTAC
CAAAAATTTTGTTCATAATTCTACGTTCGT
CGCTGAATATACTTCTGATACGACTGTAAG
ACAGGCATTGGTATTTTTTGATAAACGCCA
CGCTAGTTAAAGAAAAAGGCTGGCCGATTA
ATGTACTAGTTATTTGCAACTTGGAAGACA
TAATCTCCTTATTTTGTGTAAACAAAAACA
TGCCTTACGCTTCTTTTCTTTGACTTCAAC

L. crispatus UMB4340 | EST10 12-week void
AGAAAACAGGTATTCCAGGCGCGATAGTGG
TACCTCACACCTCTATTATTTATTTCTTTAGATCTAAACCTCATTGATCTAAAACCGTACGTTGGTACCG
CCGCAAAAGCTAATA
GTCTTACGCATGTAAACGCCGTTTTCACGG
CCGCGTTAATGTTGATTTTTCTAAATTCGA
GTTCCTAGCAGGGACAAGATTCAAGTGCTT
ACGTAAGCACCTTTTGAATTGGAGACTACT
TGATCCTATCATCTTGAAGGGCACGGGAGACAAGGCAG
TGATCCTACTGTTAACGGTGATGACTGGTATCCAGTTAG
TGATCCTACTTTGAGCTGTGTCAACGCCACAGCCGTTT
TGATCCAAGTCGTTACATCAGCCAAAAGATGAGGCTTA

L. gasseri UMB6827 | EST43 Baseline vaginal
No CRISPR

L. gasseri UMB6820 | EST43 Baseline cath
No CRISPR

L. gasseri UMB2385 | EST10 Baseline cath
No CRISPR

L. paragasseri UMB6107 | EST30 12-week void
TTCGGAGGAAAGAAATCATCCAAAGTCTT
AACTCAAACCTGTCAAGTAACCTGGCTCA
GCTCTTTTTCCCACTTAGAGCGAGCTGTA
TAGGTCTGTATTAAATACATTGCTACTCA
TTTACTTTTTGAGATTCATTAGCTCTTTC

L. paragasseri UMB6098 | EST30 12-week cath
GGAGAGTAAGCGTGAAACAAATTAAAATAATACACTAACT

CGGTCTCGTCAGCGGACTTATGCGGAA

L. paragasseri UMB6101 | EST30 12−week vaginal
GGAGAGTAAGCGTGAAACAAATTAAAATAATACACTAACT
CGGTCTCGTCAGCGGACTTATGCGGAA

L. crispatus UMB6035 | EST28 12−week void
CAGTTTAGGTACCATTTTTTGACGATCAAAATC
TGACAAGGTTGACCAAGCCGTTAAGACCATGCAA
TTAAAATAATTAAGGAGGGAACTCCAAATATCG
CAATTTCTTCCTTCGACATGTCCTTAAGAGCAT
TGACAGCTCTGCTGTAGTGATCGAATCAGACAA
TCCGACGGGGTACCCCGCGGCTGTCCATCATC
CGTGTAATGAATACGTTTAGCGTACGTATTCCG
CCAAATCTTCTAAAAATGCAATAGATATTTAGA
CAATGAAATTTTTAATGGCTGATATATTGAGTG
TGTTTCTCTAGCTGGTGGTACCAATGGTACTAA
TAAAAAAGTAATTGGTGATCCTAAAACGTATTC
TAAACGCACAGGCGTATTAGTGGGCGGACACCA
CATGTGGGCAAGGTTCAATTCCTTGCTAGTTCT
CATGGTTTCTCATTGCATCATTATCATCAACTA
TCGCTTGGTTACGGGCTAGGCAAAGCAAGCTTT
CTTGATAGCCTTGTACTTCTTTAATTGATCCAT
CACTATAACACAAAAAAAAGCCACTCCAGAGAA
TTACCCAAACCTGGAAGAGAACCCGTGTAGAAG
TCATCACGTATACTTTCACGATTGATGACAATA
CGTGTTGTTTCCATATTCATTAGATAAAACATC
TCTCTTGAAGACCATCTTTTTGGTCCTCAGTCA
TAACAATATGACCGCTACTGAATTGTTGAAGCA
TTAATGGCTGATGCAACAGCAGTCACACCATTT
CGTATAAAAAGCTAATTTGTACGCGTTTTGTTT
CATATCATGTGTTGACCAACCTAAATTTTCCAG
TAGTATTTTTTCTGTGCCTCTCTCAAGCACATT
TAAGCAAAAAGCTTTAAATATTTTCAATCAAGCT
TGGAATCATCAGAAACTAAATCAAAGAATTTTA
TACGTTTTAGGACATTCTAGAGTGACAACTGCA
CGCTTGCAGAATCCATTGAAGTCTCAATATCTT
CCATTGTCCCCGAACCAGCCTGCCGTACTGTGC
TGATTTCTTTAACCGCTTTTTGCAAAATGTCAT
ATTATTAGCAAGCTATTTATTTTCATGGAGGAG
TAGCATTGGTGCAGCAGGTTTATGGCAATTTTA
CCTACAATGATGCAAGAATGAGTAGCATGAAAG
CACTGTAATTGAAGATACAAAACTATCGTGGAA
TATGCAGAGTGGCGTGCATGTCATGATTACAGG

L. crispatus UMB6021 | EST28 12−week vaginal
CAGTTTAGGTACCATTTTTTGACGATCAAAATC
TGACAAGGTTGACCAAGCCGTTAAGACCATGCAA
TTAAAATAATTAAGGAGGGAACTCCAAATATCG
CAATTTCTTCCTTCGACATGTCCTTAAGAGCAT

TGACAGCTCTGCTGTAGTGATCGAATCAGACAA
TCCGACGGGGTACCCCGCGGCTGTCCATCATC
CGTGTAATGAATACGTTTAGCGTACGTATTCCG
CCAAATCTTCTAAAAATGCAATAGATATTTAGA
CAATGAAATTTTTAATGGCTGATATATTGAGTG
TGTTTCTCTAGCTGGTGGTACCAATGGTACTAA
TAAAAAAGTAATTGGTGATCCTAAAACGTATTC
TAAACGCACAGGCGTATTAGTGGGCGGACACCA
CATGTGGGCAAGGTTCAATTCCTTGCTAGTTCT
CATGGTTTCTCATTGCATCATTATCATCAACTA
TCGCTTGGTTACGGGCTAGGCAAAGCAAGCTTT
CCTGTAATCATGACATGCACGCCACTCTGCATA
TTCCACGATAGTTTTGTATCTTCAATTACAGTG
CTTTCATGCTACTCATTCTTGCATCATTGTAGG
TAAAATTGCCATAAACCTGCTGCACCAATGCTA
CTCCTCCATGAAAATAAATAGCTTGCTAATAAT
ATGACATTTTGCAAAAAGCGGTTAAAGAAATCA
GCACAGTACGGCAGGCTGGTTCGGGGACAATGG
AAGATATTGAGACTTCAATGGATTCTGCAAGCG
TGCAGTTGTCACTCTAGAATGTCCTAAAACGTA
TAAAATTCTTTGATTTAGTTTCTGATGATTCCA
AGCTTGATTGAAAATATTTAAAGCTTTTTGCTTA
AATGTGCTTGAGAGAGGCACAGAAAAAATACTA
CTGGAAAATTTAGGTTGGTCAACACATGATATG
AAACAAAACGCGTACAAATTAGCTTTTTATACG
AAATGGTGTGACTGCTGTTGCATCAGCCATTAA
TGCTTCAACAATTCAGTAGCGGTCATATTGTTA
TGACTGAGGACCAAAAAGATGGTCTTCAAGAGA
GATGTTTTATCTAATGAATATGGAAACAACACG
TATTGTCATCAATCGTGAAAGTATACGTGATGA
CTTCTACACGGGTTCTCTTCCAGGTTTGGGTAA
TTCTCTGGAGTGGCTTTTTTTTGTGTTATAGTG
ATGGATCAATTAAAGAAGTACAAGGCTATCAAG

L. crispatus UMB5409 | EST28 Baseline vaginal
AAAGCTTGCTTTGCCTAGCCCGTAACCAAGCGA
TAGTTGATGATAATGATGCAATGAGAAACCATG
AGAACTAGCAAGGAATTGAACCTTGCCCACATG
TGGTGTCCGCCCACTAATACGCCTGTGCGTTTA
GAATACGTTTTAGGATCACCAATTACTTTTTTA
TTAGTACCATTGGTACCACCAGCTAGAGAAACA
CACTCAATATATCAGCCATTAAAAATTTCATTG
TCTAAATATCTATTGCATTTTTAGAAGATTTGG
CGGAATACGTACGCTAAACGTATTCATTACACG
GATGATGGACAGCCGCGGGGTACCCCGTCGGA
TTGTCTGATTCGATCACTACAGCAGAGCTGTCA
ATGCTCTTAAGGACATGTCGAAGGAAGAAATTG
CGATATTTGGAGTTCCCTCCTTAATTATTTTAA
TTGCATGGTCTTAACGGCTTGGTCAACCTTGTCA
GATTTTGATCGTCAAAAAATGGTACCTAAACTG

CCTGTAATCATGACATGCACGCCACTCTGCATA
TTCCACGATAGTTTTGTATCTTCAATTACAGTG
CTTTCATGCTACTCATTCTTGCATCATTGTAGG
TAAAATTGCCATAAACCTGCTGCACCAATGCTA
CTCCTCCATGAAAATAAATAGCTTGCTAATAAT
ATGACATTTTGCAAAAAGCGGTTAAAGAAATCA
GCACAGTACGGCAGGCTGGTTCGGGGACAATGG
AAGATATTGAGACTTCAATGGATTCTGCAAGCG
TGCAGTTGTCACTCTAGAATGTCCTAAAACGTA
TAAAATTCTTTGATTTAGTTTCTGATGATTCCA
AGCTTGATTGAAAATATTTAAAGCTTTTTGCTTA
AATGTGCTTGAGAGAGGCACAGAAAAAAATACTA
CTGGAAAATTTAGGTTGGTCAACACATGATATG
AAACAAAACGCGTACAAATTAGCTTTTTATACG
AAATGGTGTGACTGCTGTTGCATCAGCCATTAA
TGCTTCAACAATTCAGTAGCGGTCATATTGTTA
TGACTGAGGACCAAAAAGATGGTCTTCAAGAGA
GATGTTTTATCTAATGAATATGGAAACAACACG
TATTGTCATCAATCGTGAAAGTATACGTGATGA
CTTCTACACGGGTTCTCTTCCAGGTTTGGGTAA
TTCTCTGGAGTGGCTTTTTTTTGTGTTATAGTG
ATGGATCAATTAAAGAAGTACAAGGCTATCAAG

L. iners UMB5018 | EST16 12-week vaginal
TCAGGATTCCCATCCTCATCTTCGGACAAATTA
TGCTATAGCGAACCATGTCCACAATGTCCTCA
TGCTGAAGTAGTCAAAGAAGTATTTAAGGCAGT
TTGGTGGTGAATTTGAATGGAACAACTGGAAC
TCTTTAGTAGCATAGCTACTTAAGTCTGACCG
GTTGTTGTAATCGTATCAAATGGCACTGAGTC

L. iners UMB5013 | EST16 12-week cath
TCAGGATTCCCATCCTCATCTTCGGACAAATTA
TGCTATAGCGAACCATGTCCACAATGTCCTCA
TGCTGAAGTAGTCAAAGAAGTATTTAAGGCAGT
TTGGTGGTGAATTTGAATGGAACAACTGGAAC
TCTTTAGTAGCATAGCTACTTAAGTCTGACCG
GTTGTTGTAATCGTATCAAATGGCACTGAGTC

L. crispatus UMB4352 | EST21 Baseline cath
AGTAGTCTCCAATTCAAAAGGTGCTTACGT
AAGCACTTGAATCTTGTCCCTGCTAGGAAC
TCGAATTTAGAAAAATCAACATTAACGCGG
CCGTGAAAACGGCGTTTACATGCGTAAGAC
TATTAGCTTTTGCGGCGGTACCAACGTACGGTTTTAGATCAATGAGGTTTAGATCTAAAGAAATAAATAA
TAGAGGTGTGAGGTA
CCACTATCGCGCCTGGAATACCTGTTTTCT
TAAGCCTCATCTTTTGGCTGATGTAACGACTTGGATCA
AAACGGCTGTGGCGTTGACACAGCTCAAAGTAGGATCA
CTAACTGGATACCAGTCATCACCGTTAACAGTAGGATCA

CTGCCTTGTCTCCCGTGCCCTTCAAGATGATAGGATCA

L. crispatus UMB4253 | EST21 Baseline vaginal
AGAAAACAGGTATTCCAGGCGCGATAGTGG
TACCTCACACCTCTATTATTTATTTCTTTAGATCTAAACCTCATTGATCTAAAACCGTACGTTGGTACCG
CCGCAAAAGCTAATA
GTCTTACGCATGTAAACGCCGTTTTCACGG
CCGCGTTAATGTTGATTTTTCTAAATTCGA
GTTCCTAGCAGGGACAAGATTCAAGTGCTT
ACGTAAGCACCTTTTGAATTGGAGACTACT
TAAGCCTCATCTTTTGGCTGATGTAACGACTTGGATCA
AAACGGCTGTGGCGTTGACACAGCTCAAAGTAGGATCA
CTAACTGGATACCAGTCATCACCGTTAACAGTAGGATCA
CTGCCTTGTCTCCCGTGCCCTTCAAGATGATAGGATCA

L. iners UMB4096 | EST12 12-week void
TTCTCGTGGCAACTTTTGCATAGGCTCATCAG
CGTGACAAGTGTAATATGTGATGATAATTATT
TCAATATCAAAGACATAAGCGAACAATTCAGG
TAGTAAATTTAGTGAGCTAGCTAAGAAGTACG
TTCAAACGCGTTTATAATGCCGCAAAAATCGT
TTAACATTAAAAATTTGCGTATTACTTGCCTT
AAGACGGTTTCGACTTTGCTCCTGATAGAAGT
CTATCTCGTCGCCTGCTTGTGCTTGTGCTCTT
AGCACGATAGAAAGAGAAAAGCGGCGGCGAAG
TCAAATGGTACTGAATCATTTGAAAAATAATA
AAAGCTGTGGGCTTTTTAGCTGATTTCTGTGC
TTTTGTTTGAAGTAAGCGTTGATTTTAGCTAT
CCAGAGAAAAGCTTGAAATGTGGCAAAGTAGG
TTAGCACTAGCAATTTGTGCTGCCCCTTTCGG
GTACTGATAAATTAGGCGACTTTGAATTGCAA
CAATTAATAAAGCTGCTAAGTTCAAAACTAAC
AGGCATATTTGGAACTGAAGTCCGCTTTTTGT
TTCTTGTAAAGCGTGTATTTTTTCGCCCTGTG

L. iners UMB4068 | EST12 12-week vaginal
TTCTCGTGGCAACTTTTGCATAGGCTCATCAG
CGTGACAAGTGTAATATGTGATGATAATTATT
TCAATATCAAAGACATAAGCGAACAATTCAGG
TAGTAAATTTAGTGAGCTAGCTAAGAAGTACG
TTCAAACGCGTTTATAATGCCGCAAAAATCGT
TTAACATTAAAAATTTGCGTATTACTTGCCTT
AAGACGGTTTCGACTTTGCTCCTGATAGAAGT
CTATCTCGTCGCCTGCTTGTGCTTGTGCTCTT
AGCACGATAGAAAGAGAAAAGCGGCGGCGAAG
TCAAATGGTACTGAATCATTTGAAAAATAATA
AAAGCTGTGGGCTTTTTAGCTGATTTCTGTGC
TTTTGTTTGAAGTAAGCGTTGATTTTAGCTAT
CCAGAGAAAAGCTTGAAATGTGGCAAAGTAGG
TTAGCACTAGCAATTTGTGCTGCCCCTTTCGG

GTACTGATAAATTAGGCGACTTTGAATTGCAA
CAATTAATAAAGCTGCTAAGTTCAAAACTAAC
AGGCATATTTGGAACTGAAGTCCGCTTTTTGT
TTCTTGTAAAGCGTGTATTTTTTCGCCCTGTG

L. iners UMB4066 | EST12 12-week cath
CACAGGGCGAAAAAATACACGCTTTACAAGAA
ACAAAAAGCGGACTTCAGTTCCAAATATGCCT
GTTAGTTTTGAACTTAGCAGCTTTATTAATTG
TTGCAATTCAAAGTCGCCTAATTTATCAGTAC
CCGAAAGGGGCAGCACAAATTGCTAGTGCTAA
CCTACTTTGCCACATTTCAAGCTTTTCTCTGG
ATAGCTAAAATCAACGCTTACTTCAAACAAAA
GCACAGAAATCAGCTAAAAAGCCCACAGCTTT
TATTATTTTTCAAATGATTCAGTACCATTTGA
CTTCGCCGCCGCTTTTCTCTTTCTATCGTGCT
AAGAGCACAAGCACAAGCAGGCGACGAGATAG
ACTTCTATCAGGAGCAAAGTCGAAACCGTCTT
AAGGCAAGTAATACGCAAATTTTTAATGTTAA
ACGATTTTTGCGGCATTATAAACGCGTTTGAA
CGTACTTCTTAGCTAGCTCACTAAATTTACTA
CCTGAATTGTTCGCTTATGTCTTTGATATTGA
AATAATTATCATCACATATTACACTTGTCACG
CTGATGAGCCTATGCAAAAGTTGCCACGAGAA

L. paragasseri UMB7013 | EST37 12-week cath
No CRISPR

L. crispatus UMB4313 | EST10 12-week cath
AGTAGTCTCCAATTCAAAAGGTGCTTACGT
AAGCACTTGAATCTTGTCCCTGCTAGGAAC
TCGAATTTAGAAAAATCAACATTAACGCGG
CCGTGAAAACGGCGTTTACATGCGTAAGAC
TATTAGCTTTTGCGGCGGTACCAACGTACGGTTTTAGATCAATGAGGTTTAGATCTAAAGAAATAAATAA
TAGAGGTGTGAGGTA
CCACTATCGCGCCTGGAATACCTGTTTTCT
TAAGCCTCATCTTTTGGCTGATGTAACGACTTGGATCA
AAACGGCTGTGGCGTTGACACAGCTCAAAGTAGGATCA
CTAACTGGATACCAGTCATCACCGTTAACAGTAGGATCA
CTGCCTTGTCTCCCGTGCCCTTCAAGATGATAGGATCA

L. crispatus UMB4315 | EST10 12-week vaginal
AGTAGTCTCCAATTCAAAAGGTGCTTACGT
AAGCACTTGAATCTTGTCCCTGCTAGGAAC
TCGAATTTAGAAAAATCAACATTAACGCGG
CCGTGAAAACGGCGTTTACATGCGTAAGAC
TATTAGCTTTTGCGGCGGTACCAACGTACGGTTTTAGATCAATGAGGTTTAGATCTAAAGAAATAAATAA
TAGAGGTGTGAGGTA
CCACTATCGCGCCTGGAATACCTGTTTTCT
TGATCCTATCATCTTGAAGGGCACGGGAGACAAGGCAG

TGATCCTACTGTTAACGGTGATGACTGGTATCCAGTTAG
TGATCCTACTTTGAGCTGTGTCAACGCCACAGCCGTTT
TGATCCAAGTCGTTACATCAGCCAAAAGATGAGGCTTA

L. paragasseri UMB4892 | EST26 Baseline vaginal
No CRISPR

L. paragasseri UMB4898 | EST26 Baseline cath
No CRISPR

L. paragasseri UMB4933 | EST20 12-week vaginal
ACAAAGCCAACTGTACCTACTAAGCCGGTTC
ACTAAACCGGTTCAACCTGCTAAACCAACTA
